# Insight of a lipid metabolism prognostic model to identify immune landscape and potential target for retroperitoneal liposarcoma

**DOI:** 10.3389/fimmu.2023.1209396

**Published:** 2023-07-06

**Authors:** Zhenyu Wang, Ping Tao, Peidang Fan, Jiongyuan Wang, Tao Rong, Yingyong Hou, Yuhong Zhou, Weiqi Lu, Liang Hong, Lijie Ma, Yong Zhang, Hanxing Tong

**Affiliations:** ^1^ First Affiliated Hospital, Anhui University of Science and Technology, Huainan, China; ^2^ Department of Laboratory Medicine, Shanghai Traditional Chinese Medicine-Integrated Hospital, Shanghai University of Traditional Chinese Medicine, Shanghai, China; ^3^ Department of General Surgery, Zhongshan Hospital, Fudan University, Shanghai, China; ^4^ Department of Pathology, Zhongshan Hospital, Fudan University, Shanghai, China; ^5^ Department of Medical Oncology, Zhongshan Hospital, Fudan University, Shanghai, China; ^6^ Department of General Surgery, Shanghai Fifth People’s Hospital, Fudan University, Shanghai, China; ^7^ Department of Liver Surgery, Renji Hospital, School of Medicine, Shanghai Jiaotong University, Shanghai, China

**Keywords:** ELOVL2, retroperitoneal liposarcoma, TCGA, lipid metabolism, immune landscape

## Abstract

**Introduction:**

The exploration of lipid metabolism dysregulation may provide novel perspectives for retroperitoneal liposarcoma (RPLS). In our study, we aimed to investigate potential targets and facilitate further understanding of immune landscape in RPLS, through lipid metabolism-associated genes (LMAGs) based prognostic model.

**Methods:**

Gene expression profiles and corresponding clinical information of 234 cases were enrolled from two public databases and the largest retroperitoneal tumor research center of East China, including cohort-TCGA (n=58), cohort-GSE30929 (n=92), cohort-FD (n=50), cohort-scRNA-seq (n=4) and cohort-validation (n=30). Consensus clustering analysis was performed to identify lipid metabolism-associated molecular subtypes (LMSs). A prognostic risk model containing 13 LMAGs was established using LASSO algorithm and multivariate Cox analysis in cohort-TCGA. ESTIMATE, CIBERSORT, XCELL and MCP analyses were performed to visualize the immune landscape. WGCNA was used to identify three hub genes among the 13 model LMAGs, and preliminarily validated in both cohort-GSE30929 and cohort-FD. Moreover, TIMER was used to visualize the correlation between antigen-presenting cells and potential targets. Finally, single-cell RNA-sequencing (scRNA-seq) analysis of four RPLS and multiplexed immunohistochemistry (mIHC) were performed in cohort-validation to validate the discoveries of bioinformatics analysis.

**Results:**

LMS1 and LMS2 were characterized as immune-infiltrated and -excluded tumors, with significant differences in molecular features and clinical prognosis, respectively. Elongation of very long chain fatty acids protein 2 (ELOVL2), the enzyme that catalyzed the elongation of long chain fatty acids, involved in the maintenance of lipid metabolism and cellular homeostasis in normal cells, was identified and negatively correlated with antigen-presenting cells and identified as a potential target in RPLS. Furthermore, ELOVL2 was enriched in LMS2 with significantly lower immunoscore and unfavorable prognosis. Finally, a high-resolution dissection through scRNA-seq was performed in four RPLS, revealing the entire tumor ecosystem and validated previous findings.

**Discussion:**

The LMS subgroups and risk model based on LMAGs proposed in our study were both promising prognostic classifications for RPLS. ELOVL2 is a potential target linking lipid metabolism to immune regulations against RPLS, specifically for patients with LMS2 tumors.

## Introduction

Retroperitoneal liposarcoma (RPLS) is a rare type of mesenchymal tumor, but the most common subtype of retroperitoneal sarcoma (1). It is also characterized by accumulation of intracellular lipid, induction of adipocyte-specific genes (2), dismal immunotherapy response and poor prognosis (3), since the significant challenges of large tumor size and adjacent organ involvement (4). In addition, other therapeutic strategies, such as combination chemotherapy and molecular targeted drugs also have limited efficacy due to intrinsic chemo-resistance, even in histology-tailored neoadjuvant chemotherapy (5). Therefore, novel strategies are needed to improve the therapeutic condition of RPLS.

Aberrant lipid metabolism and lipid metabolism reprogramming are critically involved in drug resistance (6), the adaptation of immune microenvironment (7), energy supplement, cell signaling (8) and regarded as a new hallmark of tumor ecosystem (9). Emerging evidence indicated that targeting the lipid metabolism pathway was an attractive tumor treatment strategy (10). Previous studies have indicated that lipid metabolism-associated genes (LMAGs) exhibit potent capability in predicting the prognosis of various tumors (11–14). However, lipid metabolism dysregulation in patients with RPLS remains unknown.

Immunotherapy has been extensively studied as a promising treatment, but has had limited therapeutic benefit in RPLS, which is considered as a “non-immunogenic” and highly variable tumor (15). Increasing evidence suggest that the alterations in lipid metabolism, including metabolite abundance and accumulation of lipid biomolecules, lead to local immunosuppression in the tumor microenvironment (16). However, the association between abnormality of lipid metabolism and immune microenvironment remains obscure in RPLS.

Elongation of very long chain fatty acids protein 2 (ELOVL2), an enzyme that catalyzes the elongation of fatty acids with chain lengths greater than 18 carbons. Research has shown that ELOVL2 is involved in the maintenance of cellular homeostasis in normal cells (17). Specifically, ELOVL2 was implicated in the regulation of autophagy (18) and the activity of the mTOR signaling pathway (19), which played a key role in the regulation of cell growth and proliferation. It has been suggested that the decline in ELOVL2 expression with age may contribute to the aging process and age-related diseases. Furthermore, mutations in the ELOVL2 gene have been associated with intellectual disability and developmental delay (20). However, further research was still needed to fully elucidate the functions of ELOVL2 in normal cells and its potential implications for cancer treatment.

Therefore, in this study, we explored the role of lipid metabolism dysregulation in RPLS through LMAGs related immune landscape, using multiple bioinformatics methods. A novel LMAGs based prognostic risk model was established and validated in independent cohorts. To the best of our knowledge, this is the first study to promote the understanding and clinical applications about lipid metabolism dysregulation and serve as a reliable reference for further developing target in RPLS.

## Materials and methods

### Patient and clinical specimens

The cohort-FD consists of 50 RPLS patients (34% female and 66% male) with a mean age of 55 years. In total, 50 tumor samples were surgically resected and collected in Zhongshan Hospital, Fudan University between 2018 and 2020. For scRNA-seq, four fresh surgical specimens (four primary tumors and matched PBMC) were sequenced and incorporated in further analyses. All samples were confirmed by pathologists through both cytological detections during the surgery and the paraffin section after surgery. Clinical information, including demographics and tumor clinicopathologic characteristics of all cohorts were summarized in **Supplementary Tables 1** and **2**.

### Database search

RNA transcriptome sequencing data of 150 RPLS patients and detailed characteristics were obtained from the TCGA (https://portal.gdc.cancer.gov/) and GEO (https://www.ncbi.nlm.nih.gov/geo/) databases. We excluded RPLS samples with no complete expression profile data and unknown overall survival (OS) or living status, and included clinical features such as gender, pathological grade, tumor stage and survival status in this study. Additionally, all the obtained data, TPM (transcripts per kilobase of exon model per million mapped reads) values, were normalized using the log2 (TPM + 1) transformation.

### Consensus clustering

Firstly, 135 genes were found to be associated with the prognosis of RPLS through the univariate Cox regression analysis. Consensus clustering was performed according to the expression matrix of the 135 genes using the R package “Consensus Cluster Plus”.

### Construction, validation and evaluation of risk model based on LMAGs

Least absolute shrinkage and selection operator (LASSO) analysis was performed to downsize the OS and DFS related genes previously filtrated using “glmnet” R package. The minimum lambda value was defined as the optimal value. The genes applied for the establishment of risk model was enrolled by multivariate Cox regression analysis. OS related risk score of each patient in each cohort was calculated as: OS related risk score=1.866577428*ACOT7-0.040721477*ARSJ-0.451127087*ARSK+ 0.479584604*CPT1B-0.000114123*CYP21A2 + 0.424535615*ELOVL2-1.414705024*FDX2-0.32557149*GSTM4 + 0.692304756*HACD1-0.670006158*HSD17B14-0.763286635*MTMR8-0.734212004*ORMDL2 + 1.159963024*TNFRSF21. DFS related risk score of each patient in each cohort was calculated as: DFS related risk score=0.468299088*ACOT1-0.286593499*FABP6. Patients were divided into high and low risk groups according to the medium value. ROC and Martingale residuals method were used to evaluate the predictive efficiency of model.

### Functional analysis

Differentially expressed genes (DEGs) between two LMSs were visualized using R package “Limma”. Gene Ontology (GO) analysis and Kyoto Encyclopedia of Genes and Genomes (KEGG) analysis were performed through “clusterProfifiler” R package and visualized by Metascape5 (21). Based on “GO biological process” gene dataset was downloaded from molecular signature database, Gene Set Enrichment Analysis (GSEA) was conducted to analyze the difference between subtypes. Compared with LMS2, the upregulated differential genes in LMS1 was visualized by PPI network through the Search Tool for the Retrieval of Interacting Genes (STRING) online tool and the minimum required interaction value was set as 0.7 (22).

### cBioPortal analysis

cBioPortal for cancer genomics (23) (cBioPortal, http://www.cbioportal.org, version v3.2.11) is an open-access online tool integrating the raw data from large scale genomic projects. In this study, cBioPortal was used to visualize the gene alteration of potential antigens against tumors in cohort-TCGA, including the correlation between ELOVL2 gene expression and DNA methylation.

### TIMER analysis

Tumor Immune Estimation Resource (24) (TIMER, https://cistrome.shinyapps.io/timer/) is a comprehensive resource for the systematical analysis of the immune infiltrates across diverse cancer types. In this study, TIMER was used to visualize the correlation between antigen-presenting cell (APC) infiltration and the expression of the identified potent antigens. The partial Spearman’s correlation was used to perform purity adjustment. Spearman correlation analysis was used to analyze the correlation between the abundance of APCs and the expression of the selected antigens. Statistical significance was set at *P* < 0.05.

### Construction of immune landscape

The immune score and stromal score of each sample in cohort-TCGA was calculated by the “estimate” package in R. The proportion of the 22 types of immune cells in the tumor microenvironment (TIME) of each sample was evaluated via the CIBERSORT algorithm in R software (25).

### Intra-cohort immune classifications

Unsupervised clustering of samples in each cohort was performed based on the metagene Z-score for the included populations of MCP-counter using R software, with the Euclidian distance and Ward’s linkage criterion, using the gplots package. Cohort-TCGA and Cohort-GSE30929 were further divided into 5 groups (SIC-A, B, C, D and E) (26).

### Establishment of LMAGs based nomogram

Univariate Cox regression analysis was performed to evaluate the prognostic value of identified signatures and clinicopathological features. Multivariate Cox regression analysis was used to further determine the independent prognostic factors. Two nomograms were established by the “rms” package for predicting OS and DFS. The C-index and calibration plot were constructed to estimate the accuracy and consistency of the prognostic models.

### Gene signatures for the functional orientation

The gene signatures used to determine the functional orientation were reported as previously described. Each signature was summarized as the following: immunosuppression (CXCL12, TGFB1, TGFB3 and LGALS1), T cell activation (CXCL9, CXCL10, CXCL16, IFNG and IL15), T cell survival (CD70 and CD27), regulatory T cells (FOXP3 and TNFRSF18), major histocompatibility complex class I (HLA-A, HLA-B, HLA-C, HLA-E, HLA-F, HLA-G and B2M), myeloid cell chemotaxis (CCL2), and tertiary lymphoid structures (CXCL13).

### Estimation of TLS and immune cell enrichment

12 chemokines were highly expressed by TLS, including CCL2, CCL3, CCL4, CCL5, CCL8, CCL18, CCL19, CCL21, CXCL9, CXCL10, CXCL11 and CXCL13, was applied as the gene signature of TLS. The enrichment score of TLS was calculated by single-sample gene set enrichment analysis (ssGSEA) method as implemented by R-package (27).

### Weighted gene co-expression network analysis

WGCNA algorithm was used to identify the hub genes among model genes (28). Gene co-expression modules were identified after a weighted gene co-expression network, and the association between gene network and clinical phenotype were also explored. The WGCNA-R package was applied to establish the co-expression network of all genes in the cohort-TCGA, and the genes with variance within the first 5000 were identified by the algorithm for subsequent analysis. The soft-threshold β was determined by the function “sft$powerEstimate”. The weighted adjacency matrix was transformed into a topological overlap matrix (TOM) to estimate the network connectivity, with hierarchical clustering being used to create the clustering tree structure of the TOM. Different branches of the clustering tree indicated different gene modules with different colors. Tens of thousands of genes were classified into modules based on having similar expression patterns (using their weighted correlation coefficients).

### Single-cell RNA sequencing

The single cell suspensions were converted to barcoded scRNA-seq libraries using the Chromium Single Cell 30 Library, Gel Bead & Multiplex Kit, and Chip Kit (10x Genomics), aiming for 6,000 cells per library. Samples were processed using kits pertaining to V2 barcoding chemistry of 10x Genomics. Single samples were always processed in a single well of a PCR plate, allowing all cells from a sample to be treated with the same master mix and in the same reaction vessel. For each experiment, all samples were processed in parallel in the same thermal cycler. Libraries were sequenced on an Illumina HiSeq4000, and mapped to the human genome (buildGRCh38) or to the mouse genome (build mm10) using CellRanger software (10x Genomics, version 3.0.2).

### Single-cell transcriptome data processing

The output of the cell-gene count matrix was processed with the Seurat (v 3.1.0) package of R software (version 3.6.1) for quality control and down-streaming analysis. Low-quality cells with < 200 genes or with > 40% mitochondrial genes were removed from the analysis. As the cells from tumor and adjacent normal tissues were loaded in batch for each patient, the data for each patient as individual Seurat Object. The Seurat object for each patient was integrated with the harmony algorithm (R package, Harmony, version 1.0). The top 50 principal components (PCAs) were used for graph-based clustering to identify a distinct group of cells at the indicated resolution. In the subgroup analysis, significant PCAs identified with the ElbowPlot() function were used for graph-based clustering for each cell cluster to identify subgroup cells based on the t-SNE analysis (29). The cell types of the identified cells were defined based on their expression of the canonical marker genes: T cells (CD3D, CD3E, CD4, and CD8A), B cells (MS4A1, CD79A, JCHAIN and CD19), NK cells (NCAM1, NKG7, KLRD1 and NCR1),Monocyte cells (CD14, FCN1, VCAN and CD300E), Neutrophil cells (FCGR3B, S100A9, S100A8 and CXCR1), Macrophages cells (CD163, CD68, C1QA and CSF1R), Dendritic cells (HLA-DQB2, XCR1, CD1C, and CLEC10A), Mast cells (TPSAB1, TPSB2, KIT and CPA3), Smooth muscle cells (ACAT2, TAGLN, MYL9 and MYH11), Endothelial cells (PECAM1, VWF, CLDN5 and PTPRB), Fibroblasts (DCN, COL1A1, PDPN and COLA2).

### Immunohistochemistry

Serial FFPE sections (4 um) were deparaffinized in xylene and then rehy- drated in 100%, 90% and 70% alcohol successively. Antigen unmasking was performed with a preheated epitope retrieval solution (100X citrate buffer, pH 6.0), and endogenous peroxidase was inactivated by incubation in 3% H2O2 for 20 mins. Next, the sections were preincubated with 10% normal goat serum and then incubated overnight with the following primary antibodies: anti-ELOVL2 antibody (1:50, 20308-1-AP, Proteintech); anti-CD3 antibody (1:100, ab16669, Abcam); anti-CD8 antibody (1:100, ab17147, Abcam). Next, sections were incubated with the corresponding horseradish peroxidase (HRP)-conjugated secondary antibodies (ready-to-use, MP-7451 and MP-7452, VectorLab) for 30 mins at room temperature and and development with DAB substrate (Vector Laboratories). Sections were counterstained with hematoxylin. Slides were scanned using PANNORAMIC Digital Slide Scanners, and QuPath software was used to quantify positive staining cells.

### Multiplexed immunohistochemistry

mIHC was performed in four DDLPS cases of cohort-sc-RNA seq. Briefly, Fresh tumor tissues were fixed in 4% paraformaldehyde solution and embedded in paraffin. FFPE slides were made using 4 μm sections of the tumor samples. Deparaffinization and rehydration were performed with xylene and ethanol respectively, followed by microwave antigen retrieval using heated citric acid buffer (pH 6.0) for 10 minutes and endogenous peroxidase blocking in 3% H2O2 for 20 minutes. Blocker/Diluent was used to block nonspecific binding sites. Afterward, relevant primary antibodies were incubated for 1 hour at room temperature, followed by the corresponding secondary antibodies for 30 minutes. Slides were then incubated with fluorescein TSA plus for 10 minutes (Akoya Boscience, NEL861001KT), after which microwave antigen retrieval was repeated with the above steps until the last antibody was added. After multiplexing, DAPI (Sigma, D9542) was used to stain the nuclei. Antibodies and fluorescent dyes used for multiplexing are listed in **Supplementary Table 3**. The slides were scanned by Vectra 3 automated high-throughput multiplexed biomarker imaging system (Akoya Phenoimager HT). Immune cells were classified into the following types: T cells (CD3^+^), B cells (CD20^+^), DC cells (CD11b^+^), NK cells (CD57^+^), Mac (CD68^+^) and ELOVL2^+^ cell.

### Statistical analysis

SPSS 22.0 (SPSS Inc., Chicago, IL, USA) and R 4.0.4 (R Foundation for Statistical Computing, Vienna, Austria; http://www.r-project.org/) were used for all statistical analyses. Univariate and multivariate Cox regression analyses, ROC curve analysis and K-M survival analysis were performed by R software and the corresponding R packages. The continuous data are expressed as the mean ± standard deviation (SD). The Wilcoxon test was used for comparisons between the two groups, and the Kruskal-Wallis test was used for comparisons of prognosis between groups. Except for the special instructions, all statistical tests were two-tailed, and a *P* < 0.05 was considered to be statistically significant.

## Results

### Identification of multi-omics landscape and prognostic LMAGs in RPLS

The whole study process, including consensus clustering, immune landscape visualization, DEGs analysis, nomogram construction and WGCNA analysis were systematically evaluated and depicted in the workflow chart, as shown in **Supplementary Table 4**.

**Figure 1:**
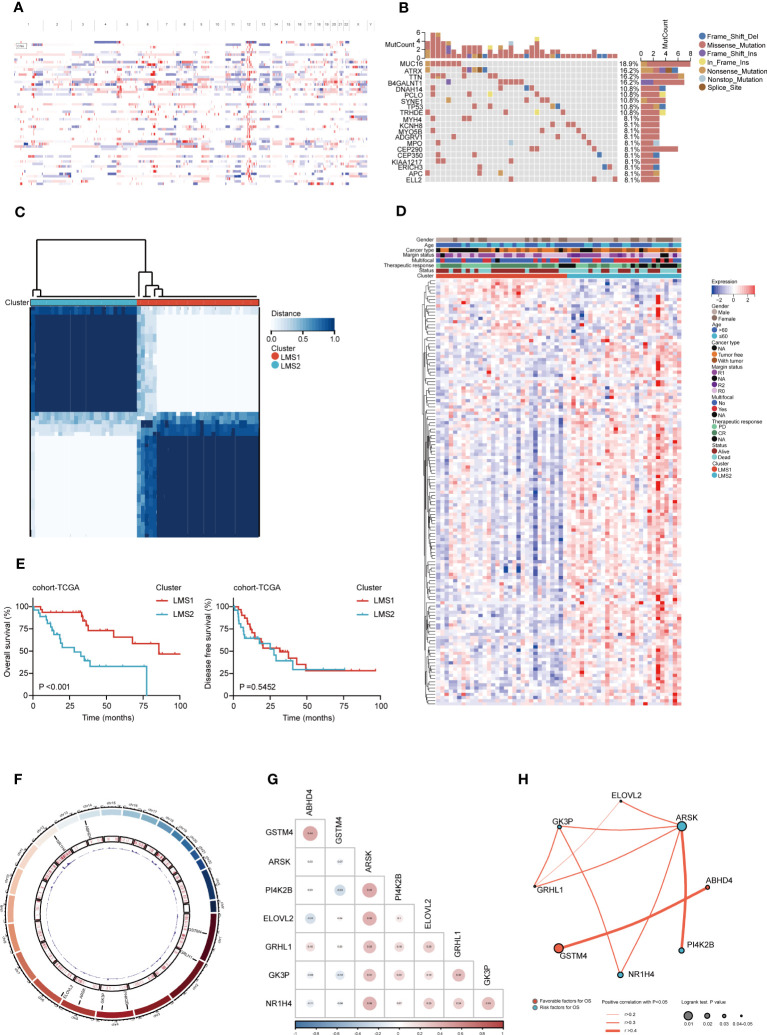
Identification of multi-omics landscape and prognostic LMAGs in RPLS. **(A)** The chromosomal distribution of the aberrant copy number genes in RPLS of cohort-TCGA. **(B)** Genetic profile of cohort-TCGA. **(C)** Unsupervised clustering of cohort-TCGA based on the LMAGs. **(D)** Heatmaps integrating 135 LMAGs and associated clinicopathologic features in cohort-TCGA. **(E)** Kaplan-Meier survival curve of OS and DFS for LMSs in cohort-TCGA. **(F)** Circos plot depicting the location on chromosomes and expression level of 8 overlapping OS-related and DFS-related LMAGs. **(G)** Triangle heatmap showing the correlation features among 8 overlapping LMAGs in cohort-TCGA. **(H)** Correlation and prognosis analysis showing the inter-gene communications among eight LMAGs.

To systematic appraise the lipid metabolism dysregulation in RPLS, 741 LMAGs were obtained from the MSigDB database. Initially, we encapsulated the incidence of copy number variations and somatic mutations in cohort-TCGA, where 14862 amplified genes were screened to identify potent antigens (**Figure 1A**), while missense mutation was the most common variant classification (**Figure 1B**). Additionally, we investigated the incidence of CNV among these LMAGs. By evaluating the frequency of CNV status, existing CNV alterations of LMAGs were also clarified, and the top 35 genes in amplified or deleted CNV status were summarized (**Supplementary Figure 1A**).

Subsequently, according to the transcriptomic data, consensus clustering was performed to cluster RPLS patients into two lipid metabolism subgroups (LMSs) (K = 2) (**Figure 1C**) based on 135 overall survival (OS) related genes by univariable Cox analysis (**Supplementary Figure 1B**). 31 and 27 patients were clustered into LMS1 and LMS2, respectively (**Supplementary Figure 1C**). Heatmap visualization also indicated that prognostic LMAGs profiles differed significantly between LMS1 and LMS2, while LMS2 was enriched with LMAGs (**Figure 1D**), but predicted poor prognosis (**Figure 1E**). Interestingly, two subtypes harbored heterogenous somatic mutations profiles, demonstrated ATRX and B4GALNT1 as the genes with the highest mutation frequency in LMS1, but MUC16 in LMS2 (**Supplementary Figures 1D, E**). However, not significant difference was found in genome altered fraction and mutation counts between LMS1 and LMS2 (**Supplementary Figures 1F, G**).

Moreover, we also investigated 26 disease free survival (DFS) related LMAGs through univariate Cox analysis (**Supplementary Figure 1H**). Intersecting the results of OS-related and DFS-related genes, 8 overlapping LMAGs (GSTM4, GRHL1, PI4K2B, GK3P, ARSK, ELOVL2, NR1H4, and ABHD4) were excavated and eligible for further screen prognostic relevant antigens (**Supplementary Figure 1I**). The location on chromosomes and expression levels were visualized in **Figure 1F**. The regulatory network described the comprehensive landscapes of the 8 LMAGs indicating their interactions, correlation feature (**Figure 1G**) and prognostic values (**Figure 1H**). These findings indicated that the LMAGs classified RPLS patients into two subtypes with different molecular features and prognosis.

### Heterogeneous functional enrichment and immune landscape in LMAGs subtypes

**Figure 2:**
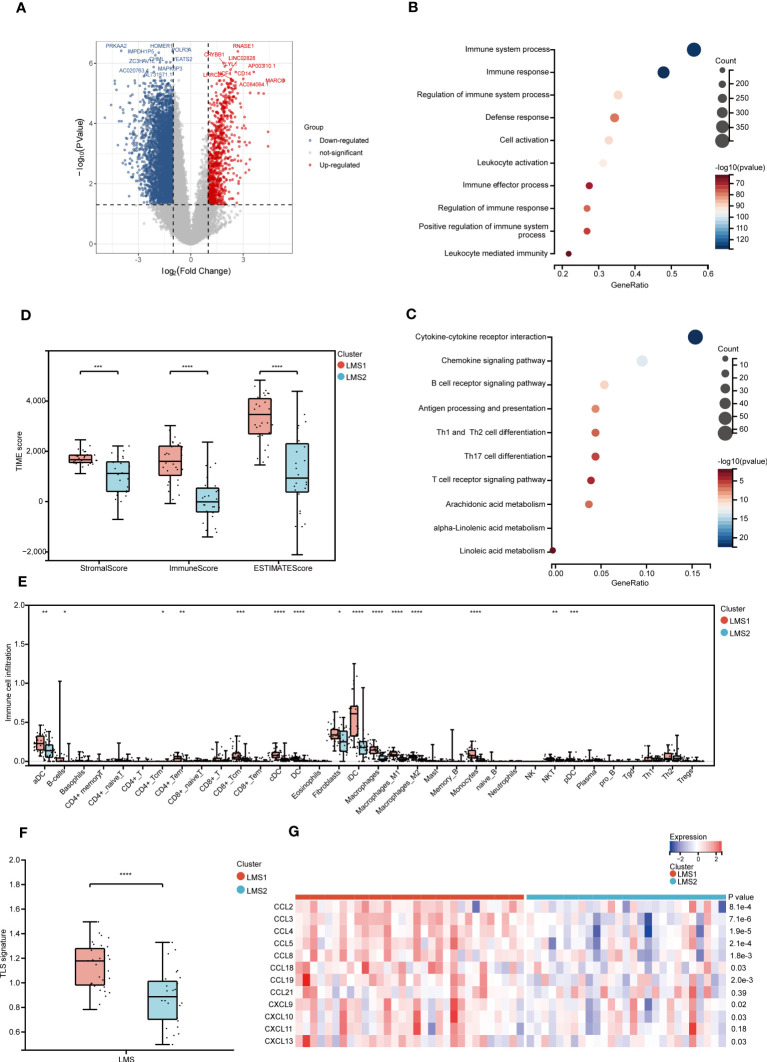
Heterogeneous functional enrichment and immune landscape in LMAGs subtypes. **(A)** Volcano plot depicting the gene expression difference between LMS1 and LMS2. **(B, C)** Bubble diagram showing the biological processes enriched by GO analysis and the signaling pathways enriched by KEGG analysis. **(D)** The comparison of stromal score, immune score and ESTIMATE score calculated by ESTIMATE algorithm. **(E)** Abundance of immune-related molecular signatures evaluated by XCELL indicating significant differences between LMS1 and LMS2. **(F)** Estimation of TLS based on 12-chemokine signature between LMS1 and LMS2. **(G)** Heatmap showing the transcriptomic expression of 12-chemokine involving TLS according to LMSs. ns, not significant, *P < 0.05, **P < 0.01, ***P < 0.001, and ****P < 0.0001.

To better understand the innate difference of survival and underlying signaling mechanisms between LMS1 and LMS2, DEGs and functional enrichment analyses were performed, respectively. A total of 4144 DEGs were identified, of which 3586 genes were downregulated and 558 genes were upregulated in LMS2, as compared with LMS1 (**Figure 2A**). GO enrichment analysis indicated that these up-regulated DEGs were involved in positive regulation of immune system process, immune response and regulation of immune system process (**Figure 2B**). Similarly, KEGG enrichment analysis also validated these pathways associated with cytokine-cytokine receptor interaction, chemokine signaling pathway and B cell receptor signaling pathway, of which are part of the immune response (**Figure 2C**). Meanwhile, PPI analysis further confirmed 15 submodels, all of which were closely associated with immune and metabolism (**Supplementary Figure 2A**). These results depict that the expression of LMAGs is closely related to immune-related biological processes, confirming that lipid metabolic reprogramming is significantly associated with tumor immune microenvironment (TIME) in RPLS.

To further evaluate the dysregulation of immune and metabolism remodeling involved in RPLS, a series of TIME profiles was conducted. Firstly, we illustrated the distribution feature of previously reported six pan-cancer immune subtypes (C1-C6) (30), of which LMS1 and LMS2 were mostly clustered into C1 (Wound Healing), C2 (IFN-γ Dominant), C3 (Inflammatory), C4 (Lymphocyte Depleted) and C6 (TGF-β Dominant) (**Supplementary Figure 2B**). Intriguingly, C3 and C6 presented a higher proportion in LMS1, while they were associated with better outcomes. Accordingly, immune score, stromal score and ESTIMATE score were also calculated using the ESTIMATE algorithm (**Figure 2D**). Remarkably, LMS1 demonstrated significantly higher TIME scores and better prognosis than those in LMS2, implying that favorable immune components and immune-related molecules were abundant in LMS1.

Next, the relationship between LMSs and 34 infiltrated immune cells was further explored. In concordance with previous TIME scores, LMS1 harbored more infiltrated DC, B cells, CD4+Tem, macrophages, monocytes and NKT than those in LMS2 (**Figure 2E**), indicating the significant impacts of these immune cells in the progression of RPLS. However, the complete immune response involves a close combination of multiple events, not only the infiltrated immune cells (4). Thereafter, we further calculated and compared the immune activity score of each step through TIP analysis. Similarly, the abundance of anti-tumor immune cells was significant higher in LMS1 than those in LMS2, as well as in Step1, Step3 and Step5 (**Supplementary Figure 2C**). In addition, we explored substantial differences in TLS-associated 12-chemokine signature between LMS1 and LMS2 (**Figure 2F**), while the expression of lymphoid-structures-associated B-cell-specific chemokine CXCL13 was notably higher in LMS1 (**Figure 2G**).

Accumulating evidence indicated that tumor with high TMB level predicted better efficacy of immunotherapy (31). We then estimated the value of TMB in both LMS1 and LMS2, but not significant difference was found (**Supplementary Figure 2D**). Intriguingly, patients in LMS1 with low TMB demonstrated a satisfactory survival benefit. Considering the importance of immune checkpoint inhibitors in the treatment of solid tumor (3), we further examined the differences in immune checkpoint profiles and found notably substantial differences in CD28, CD40, CD86, HAVCR2 and PD-1, between these two subtypes (**Supplementary Figure 2E**). Immunogenic cell death (ICD) has been classified as a form of regulated cell death (RCD) that is sufficient to activate an adaptive immune response (32). We next identified ICD-related genes and analyzed the expression patterns. Importantly, we discovered that significant higher expression of FPR1, TLR4 and CXCL10 were enriched in LMS1 (**Supplementary Figure 2F**). Taken together, these findings demonstrated the unique characteristics of TIME within two LMSs, offering a conducive complement to previous studies.

### Identification of immune gene co-expression modules and immune hub genes of RPLS

**Figure 3:**
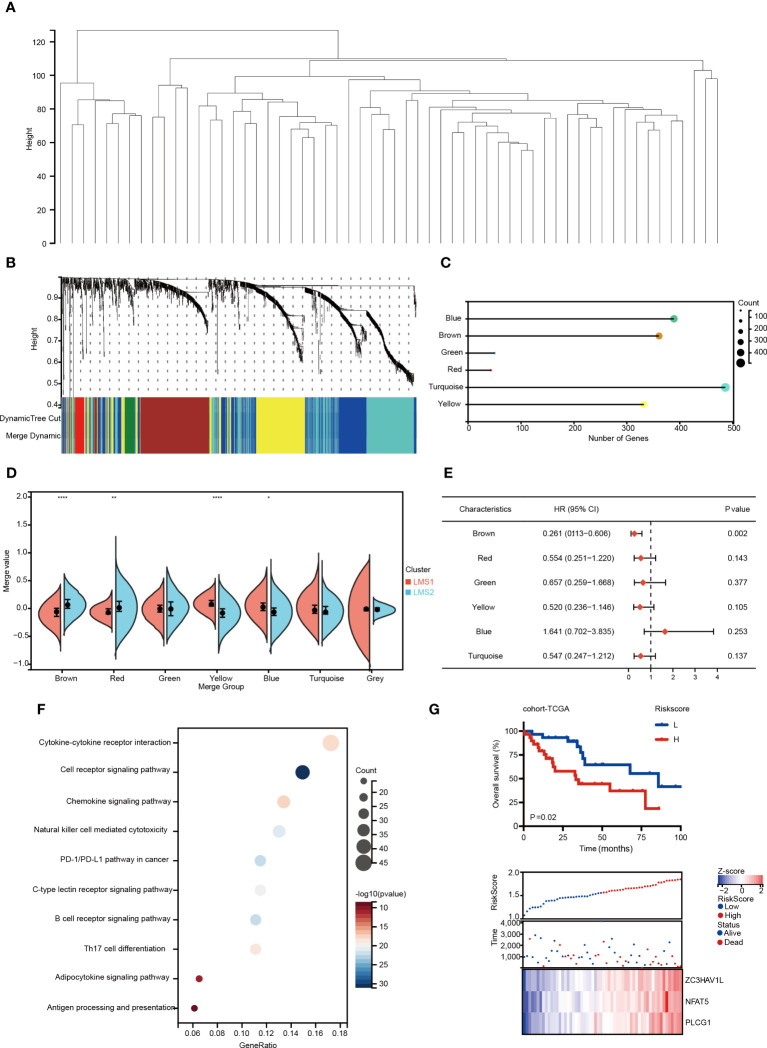
Identification of immune gene co-expression modules and immune hub genes of RPLS. **(A, B)** Gene co-expression network analysis based on the immune-related genes. **(C)** Dot plot of the co-expression gene modules. **(D)** The comparison of identified gene modules according to LMSs. **(E)** Forest maps depicting prognosis prediction value of six modules in RPLS. **(F)** Bubble plot showing the top 10 KEGG terms enrichment pathways from the brown module. **(G)** Kaplan-Meier survival curve of OS for two risk-score groups in cohort-TCGA. Distribution of survival status according to risk score in RPLS. Heatmap illustrating the expression panel of three immune hub genes according to the risk score. *P < 0.05, **P < 0.01, and ****P < 0.0001.

The immune gene co-expression module was designed and applied to classify immune-related genes, whose expression may significantly influenced the effectiveness of potential targets (33). Therefore, we re-analyzed and enrolled immune-related genes to establish gene modules through WGCNA (**Figure 3A**). The soft threshold was set at four in the scale-free network (**Supplementary Figure 3A**). The representation matrix was converted to adjacency and next to a topological matrix. The average-linkage hierarchy clustering approach was applied with a minimum of 30 genes for each network according to the standard of a hybrid dynamic shear tree. Eigengenes of each module were calculated and the close modules were integrated (height = 0.25, deep split = 3 and min module size = 30). Notably, six gene modules were identified (**Figures 3B, C**), and correlation feature was also visualized (**Supplementary Figure 3B**). In addition, the module eigengenes in LMS1 were significantly higher in yellow and blue modules (**Figure 3D**). Moreover, the prognostic analysis indicated that eigengenes in the brown module was significantly associated with OS in RPLS (**Figure 3E**).

Further functional enrichment analysis illustrated that genes involved in cytokine-cytokine receptor interaction and T cell receptor signaling were enriched in the brown module (**Figure 3F**). The brown module (361 immune-related genes) was further selected, and three of them (PLCG1, ZC3HAV1L and NFAT5) were filtered to build the risk score through LASSO algorithm (**Supplementary Figures 3C, D**). Patients were classified into the high-risk and low-risk groups, while high-risk group predicted unfavorable OS (*P* = 0.02) (**Figure 3G**). The area under the receiver operating curve (AUC) was 0.75, indicating a good accuracy of the model (**Supplementary Figure 3E**). Taken together, this risk model may serve as a novel tool for prognostic predicting in RPLS, based on the immunogenic genes co-expression network.

### Development of survival and relapse risk models and nomograms based on LMAGs

Given the significant biological roles of LMAGs in lipid metabolic reprogramming, the association between LMAGs-related risk score and the prognosis needed thoroughly study. Thus, two prognostic models were conducted for OS and DFS, respectively.

**Figure 4:**
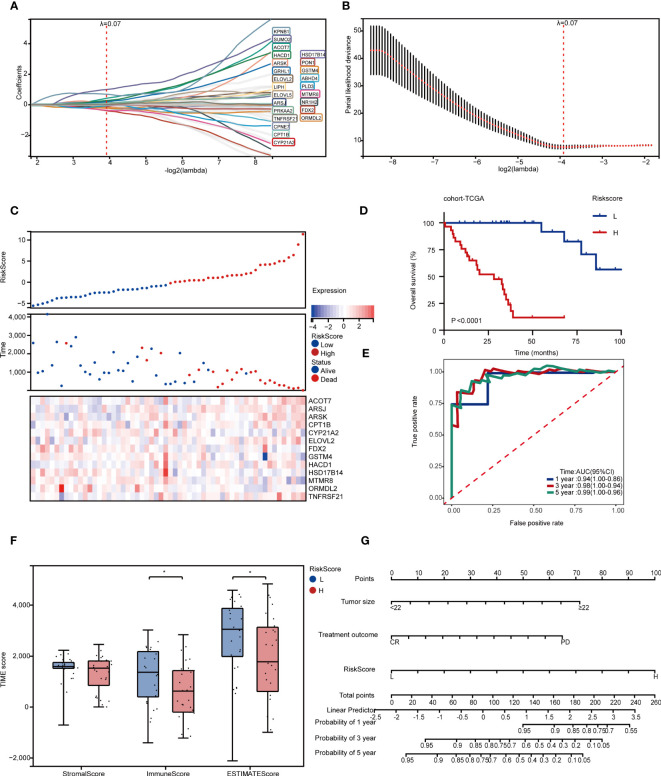
Development of survival and relapse risk models and nomograms based on LMAGs. **(A, B)** LASSO regression analyses for screening optimal OS-related LMAGs. **(C)** Distribution of survival status according to risk score in RPLS. Heatmap illustrating the expression panel of 13 OS-related LMAGs according to the risk score. **(D)** Kaplan-Meier survival curve of OS for two risk-score groups in cohort-TCGA. **(E)** Time-dependent ROC curve analyses of the LMAGs risk signature in cohort-TCGA. **(F)** The comparison of stromal score, immune score and ESTIMATE score calculated by ESTIMATE algorithm in two risk-score groups. **(G)** Nomogram predicting OS for RPLS. *P < 0.05.

24 LMAGs were found to be considerably linked to the OS of patients through LASSO regression analysis (**Figures 4A, B**), 13 of which were tested and selected for the risk score model from multivariate Cox analysis (**Supplementary Figure 4A**). We also investigated the relationship between risk score and survival status, and the low-risk subgroup harbored significant more alive statuses (**Figure 4C**) and better OS than those in high-risk subgroup (**Figure 4D**). Specifically, this OS related model indicated a great accuracy with AUC values of 0.94 in 1 year, 0.97 in 3 years and 0.97 in 5 years (**Figure 4E**).

To evaluate and validate the universality of this LMAGs-related prognostic model from cohort-TCGA, an independent dataset (cohort-FD) was performed as a validation cohort. The risk score of each case in cohort-FD was calculated using the same formula as that for the cohort-TCGA. Similarly, patients in high-risk group suffered unfavorable OS than those in low-risk group (**Supplementary Figure 4B**). In addition, the AUC values of this model according to ROC analysis were 0.7 in 1 year, 0.8 in 3 years, and 0.85 in 5 years (**Supplementary Figure 4C**).

Since this model predicted great potency in clinical prognosis based on LMAGs, we further investigated the correlation between risk score and TIME in cohort-TCGA. In concordance with previous findings, the high-risk subgroup was characterized with a significantly lower immune score (**Figure 4F**), and infiltrated less CD8+ T cells and plasma cells, but more resting memory CD4+ T cells and Tregs than those in low-risk subgroup (**Supplementary Figure 4D**), implying that high-risk subgroup was considered with the characteristics of immunosuppressive status.

Meanwhile, we also explored the value of model between the two subgroups stratified by different clinical features. Univariate Cox analysis indicated that patients with dismal OS were authenticated with larger tumor size, chemotherapeutic efficacy and high risk score (**Table 1**). Multivariate Cox analysis further confirmed that all of them were independent risk factors (**Table 1**). Subsequently, we developed a nomogram for OS prediction using these two clinical parameters and the LMAGs-based risk scores. A calibration plot for internal validation of the nomogram presented excellent consistency between the nomogram-predicted probability and actual observations of the 1-, 3-, and 5-year OS (**Figure 4G**).

**Table 1:** Univariate and multivariate analysis of OS in cohort-TCGA (n=58).

Variables	Univariate analysis		Multivariate analysis	
	HR (95%CI)	P value	HR (95%CI)	P value
Age, years (>60 vs. ≤60)	3.778 (0.888-16.075)	0.072		
Sex (female vs. male)	0.531 (0.240-1.176)	0.119		
Tumor size, cm (>20 vs. ≤20)	2.579 (1.089-6.106)	**0.031**	5.299 (1.302-21.567)	**0.02**
Multifocal (single vs. multiple)	2.059 (0.899-4.715)	0.088		
Residual tumor (yes vs. no)	2.194 (0.909-5.299)	0.081		
Treatment outcome (CR vs. PD)	0.170 (0.047-0.611)	**0.007**	0.220 (0.052-0.935)	**0.04**
Recurrence (yes vs. no)	2.388 (0.953-5.983)	0.063		
Riskscore (high vs. low)	26.477 (5.915-118.520)	**<0.001**	10.296 (1.960-54.073)	**0.006**

Bold values identify statistical significance (p < 0.05).

OS, Overall Survival; HR, hazard ratio; CI, confidential interval; CR, Complete Response; PD, Progressive Disease.

Variables with P < 0.05 in the univariate analysis were included in the multivariate analysis.

Given the significant high local recurrence rate in clinical treatment of RPLS (34), 21 LMAGs were also found to be considerably linked to the DFS of patients through LASSO regression analysis (**Supplementary Figure 4E**), 2 of which were tested and selected for the prediction model in the multivariate Cox analysis (**Supplementary Figure 4F**). The association between risk score and recurrence status was next evaluated, and the low-risk subgroup harbored significant more alive statuses (**Supplementary Figure 4G**) and better DFS than those in high-risk subgroup (**Supplementary Figure 4H**). Similarly, this DFS related model also presented a great accuracy with AUC values of 0.72 in 1 year, 0.89 in 3 years and 0.79 in 5 years (**Supplementary Figure 4I**). In addition, univariate Cox analysis indicated that patients with worse DFS were authenticated with tumor residue, poor chemotherapeutic efficacy and high risk score (**Table 2**). Multivariate Cox analysis further confirmed that dismal chemotherapeutic efficacy and high risk score were independent risk factors (**Table 2**). Furthermore, a nomogram for DFS prediction was conducted with excellent consistency (**Supplementary Figure 4J**).

**Table 2:** Univariate and multivariate analysis of DFS in cohort-TCGA (n=58).

Variables	Univariate analysis		Multivariate analysis	
	HR (95%CI)	P value	HR (95%CI)	P value
Age, years (>60 vs. ≤60)	2.686 (0.941-7.666)	0.065		
Sex (female vs. male)	1.217 (0.561-2.642)	0.619		
Tumor size, cm (>20 vs. ≤20)	1.619 (0.747-3.5008)	0.222		
Multifocal (single vs. multiple)	1.594 (0.766-3.319)	0.213		
Residual tumor (yes vs. no)	2.169 (1.017-4.626)	**0.045**	1.880 (0.740-4.779)	0.185
Treatment outcome (CR vs. PD)	0.109 (0.045-0.265)	**<0.001**	0.144 (0.056-0.372)	**<0.001**
Riskscore (High vs. Low)	3.662 (1.748-7.670)	**<0.001**	2.854 (1.088-7.491)	**0.033**

Bold values identify statistical significance (p < 0.05).

DFS, disease free survival; HR, hazard ratio; CI, confidential interval; CR, Complete Response; PD, Progressive Disease.

Variables with P < 0.05 in the univariate analysis were included in the multivariate analysis.

### Identification of lipid metabolism-associated targets

**Figure 5:**
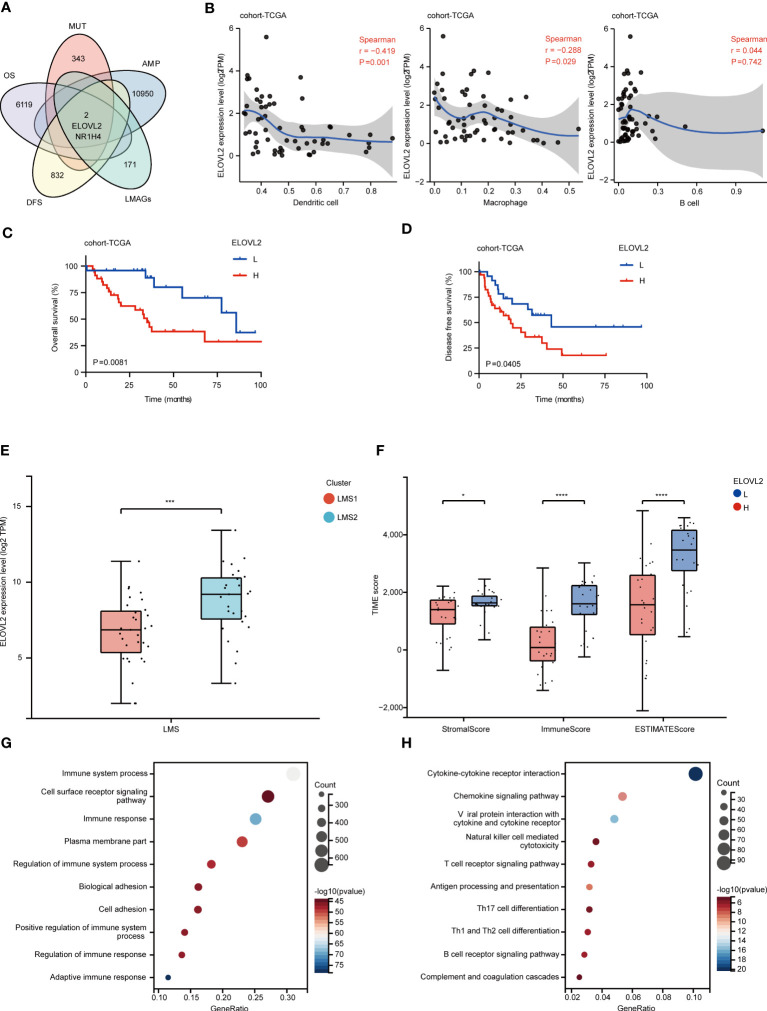
Identification of lipid metabolism-associated targets. **(A)** The intersection of amplification, mutation, OS-related and DFS-related genes were identified as potential lipid metabolism-associated targets. **(B)** Identification of targets associated with APCs. Correlation between ELOVL2 expression and infiltration of macrophages, dendritic cells and B cells in RPLS. **(C, D)** Kaplan-Meier survival curves of OS and DFS for ELOVL2 expression in cohort-TCGA. **(E)** The comparison of ELOVL2 transcriptomic expression in LMS1 and LMS2. **(F)** The comparison of stromal score, immune score and ESTIMATE score calculated by ESTIMATE algorithm according to ELOVL2 expression. **(G, H)** Bubble diagram depicting the biological processes enriched by GO analysis and the signaling pathways enriched by KEGG analysis according to upregulated DEGs in ELOVL2^high^ group, compared with ELOVL2^low^ group. *P < 0.05, ***P < 0.001, and ****P < 0.0001.

To explore key genes that functioned as potential candidates for RPLS, we further systematically screened and identified two candidates (NR1H4 and ELOVL2) with both gene amplification and mutation, which were also associated both OS and DFS from the 135 LMAGs (**Figure 5A**). Given the essential role of antigen-presenting cells (APCs) in the function of immunological reaction, we also evaluated the relationship of these two genes with APCs using TIMER analysis (35). Intriguingly, ELOVL2, but not NR1H4, was identified with closely related to APCs (Pearson correlation coefficient > 0.3; **Figure 5B**; **Supplementary Figure 5A**), which could serve as a potential target and triggered strong immune response. In addition, similar results were also found in TCGA-SARC (**Supplementary Figure 5B**). Notably, survival analysis demonstrated that high mRNA expression of ELOVL2 was associated with unfavorable OS and DFS (**Figures 5C, D**), suggesting ELOVL2 was of importance in RPLS development and progression. In concordance with cohort-TCGA, the mRNA expression of ELOVL2 validated similar prognostic efficiency in both cohort-FD and cohort-GSE30929 (**Supplementary Figures 5C, D**).

Dedifferentiated liposarcoma (DDLPS) was often progressed from primary or recurrent well-differentiated liposarcomas (WDLPS), which constituted the most common pathological type of RPLS (36). Thus, we also investigated the heterogeneous expression of ELOVL2 in WDLPS and DDLPS. Interestingly, the expression of ELOVL2 was significantly higher in LMS2 than that in LMS1 (**Figure 5E**). Specifically, ELOVL2 exhibited a potential enrichment in DDLPS, as compared with WDLPS in both cohort-FD and cohort-GSE30929 (**Supplementary Figures 5E, F**), indicating the significant impacts of ELOVL2 in the dedifferentiation evolution of RPLS.

To further identify the association between immune status and ELOVL2 expression, a series of TIME profiles was also conducted. Accordingly, immune score, stromal score, ESTIMATE score (**Figure 5F**), infiltrating immune cells and TLS signature (**Supplementary Figures 6A, B**) were all calculated. As expected, we discovered that significant difference was found in the immune status between ELOVL2^high^ and ELOVL2^low^ subgroups, suggesting that RPLS patients with high expression of ELOVL2 might towards a status of immune desert. In addition, GO enrichment analysis indicated that high expression of ELOVL2 was involved in immune system process and cell surface reportor signaling. Similarly, KEGG enrichment analysis also validated cytokine-cytokine receptor interaction and cytokine signaling pathway (**Figures 5G, H**).

Accumulating evidence indicated that the increase of DNA methylation of ELOVL2 leaded to the decrease of its protein expression and polyunsaturated fatty acid synthesis, but the accumulation of short chain fatty acids, which is closely related to aging (37). Thus, we investigated the genome and epigenome landscape of ELOVL2 in cohort-TCGA (**Supplementary Figure 6C**), and 13 ELOVL2 related CpG sites were identified. However, only the cg20462795 exhibited significantly survival patterns (**Supplementary Figure 6D**), depicting that ELOVL2 associated epigenetic metabolic axis could be a novel therapeutic target in RPLS.

Transcription factor (TF) is one of the most common tool that involving in regulating gene expression (38). Thereafter, we further systematically screened and identified three ELOVL2 related TFs (TFDP1, TP73 and MYBL2) in TCGA-SARC cohort (**Supplementary Figure 6E**). Interestingly, high expression of them were significantly associated with decreased OS (**Supplementary Figure 6F**). Consistent with survival prediction occurring in our cohort, TFDP1 and MYBL2 exhibited similar survival patterns in both TCGA-SARC and GTEx databases (data not shown). Taken together, these results demonstrated that gene expression was determined by the synergistic regulation of both transcription and epigenetic factors.

### ELOVL2 dominated lipid metabolism reprogramming and executive TIME affect prognosis in RPLS

Considering the dysregulation of immune status and metabolism remodeling involved in RPLS, we discovered that ELOVL2 played an essential role in tumor progression and even dedifferentiation, and served as the only one lipid metabolism related target biomarker. Moreover, PLCG1, ZC3HAV1L and NFAT5 were selected as immune hub genes through immune gene co-expression modules analysis and predicted great prognostic efficacy. Therefore, we re-analyzed the association between ELOVL2 and three hub genes.

**Figure 6:**
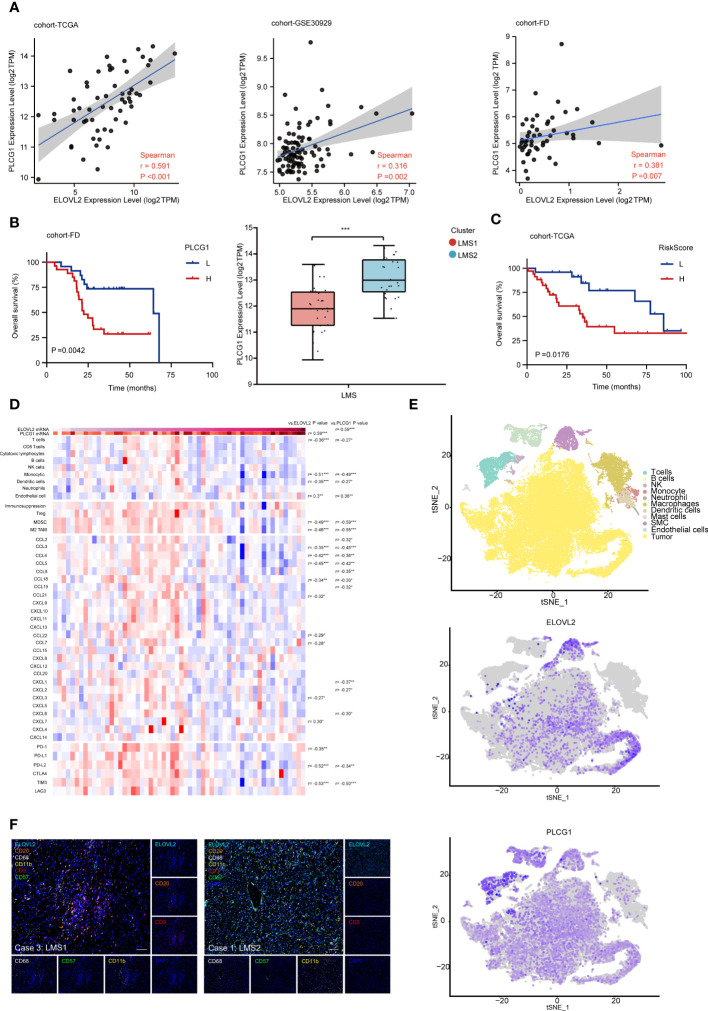
ELOVL2 dominated lipid metabolism reprogramming and executive TIME affect prognosis in RPLS. **(A)** Correlation among the transcriptomic expression of ELOVL2 and PLCG1 in three databases (cohort-TCGA, GSE30929 and FD). Spearman’s correlation coefficient was calculated. **(B)** Kaplan-Meier survival curves of OS for PLCG1 expression in cohort-FD. The comparison of PLCG1 transcriptomic expression in LMS1 and LMS2. **(C)** Kaplan-Meier survival curve of OS for combined riskscore of ELOVL2 and PLCG1 in cohort-TCGA. **(D)** Heatmap showing the transcriptomic expression of genes involving TIME according to ELOVL2 and PLCG1 expressions. The histogram on the right showing the results of differential expression analysis of each gene in indicated comparisons on the top of each column. **(E)** t-SNE plot showing of the overview of 11 cell clusters in the integrated single-cell transcriptomes of 54126 cells from four RPLS. Clusters are named as indicated cell subsets according to the specific gene expression patterns, color-coded according to ELOVL2 and PLCG1. **(F)** Representative mIHC images show the positivity of CD3, CD8, CD20, CD11b, CD68, and ELOVL2 in case 3 and case 1. Scale bar, 100 um. *P < 0.05, **P < 0.01, and ***P < 0.001.

Firstly, we validated these hub genes in cohort-FD and cohort-GSE30929 through survival analysis. Interestingly, the expression of PLCG1 was positive associated with ELOVL2 in all cohorts (**Figure 6A**). In addition, PLCG1 was significantly higher in LMS2 than that in LMS1, and exhibited similar survival patterns in all cohorts (**Figure 6B**). Next, the combined prognostic analysis of ELOVL2 and PLCG1 was also performed. Patient stratification based on these three groups presented that the Group I was associated with favorable prognosis, whereas the Group III was associated with dismal prognosis, and Group II was associated with medium prognosis (**Supplementary Figure 7A**). Subsequently, we developed another prognostic model for OS prediction based on ELOVL2 and PLCG1, which suggested a great prognostic efficacy (**Figure 6C**).

In order to systematically comprehend the extent of relevancy between ELOVL2 and PLCG1 within TIME, we re-analyzed the expression profile in cohort-TCGA. Based on comparative analysis, we observed remarkable positive correlation between ELOVL2 and PLCG1. In addition, the infiltration of T cells, monocytics, dendritic cells, MDSC and M2-TAM were significantly lower with high expression of ELOVL2 and PLCG1. Moreover, the chemokine enrichment of CCL3, CCL4, CCL5 and CCL18, as well as PDCD1LG2 and HAVCR2 were significantly negative associated with high expression of ELOVL2 and PLCG1 (**Figure 6D**). These results demonstrated that highly expression of ELOVL2 and PLCG1 in TIME of RPLS was involved with a immune-exculded phenotype.

To fully characterize the specific cellular localization ELOVL2 and PLCG1 in RPLS, we first evaluated them in the human protein atlas and further validated them *in situ* single cell spatial phenotype analysis through single cell RNA-sequencing and immunohistochemistry (IHC) in cohort-FD. Interestingly, ELOVL2 was seemed enriching in fibroblasts (no images in database), while PLCG1 in T cells (**Supplementary Figure 7B**). At the single-cell level, ELOVL2 was expressed in tumor cells, cancer associated fibroblasts (CAFs) and smooth muscle cells (SMCs), while PLCG1 was presented in CD4+ and CD8+ T cells, as expected (**Figure 6E**; **Supplementary Figure 7C**). According to the analysis of bulk-RNA seq data, case 1/2 was defined as LMS2 and case 3/4 with LMS1, respectively. Next, mIHC was performed to validate previous findings in these four DDLPS. Consistent with RNA-seq data,

CD3^+^T cell, CD20^+^B cell, CD11b^+^DC cell and CD68^+^ macrophages presented a higher infiltration in case3 and case4, but less ELOVL2^+^ cells. On the contrary, less CD3^+^T cells, CD20^+^B cells and CD68^+^ macrophages, but more ELOVL2^+^ cells were found in case 1 and case 2 (**Figure 6F**; **Supplementary Figure 7E**). Moreover, the densities of CD3^+^ and cytotoxic CD8^+^ T cells in the area of tumor and invasive margin were quantified by IHC. We observed that the density of CD3^+^ and cytotoxic CD8^+^ T cells were significantly but negatively associated with ELOVL2^+^ cells (**Supplementary Figure 7D**). In concordance with previous findings, the mRNA expression level of CD3 and CD8 was negatively associated with ELOVL2 (**Supplementary Figure 7F**). Taken together, the dysregulation of lipid metabolism might remodel an immune desert of TIME further supported their crucial roles in the evolution of RPLS.

## Discussion

RPLS is one of the most aggressive malignancies with a heterogeneous molecular profile, lipid metabolism dysregulation, limited medical efficacy and highly local recurrence rate (39). The combination of doxorubicin and ifosfamide is the first-line option in treating advanced RPLS, with limited clinical benefits and a median OS of just 8 to 14 months (40). Although immunotherapy has revolutionized oncology from the therapeutic point of view, its effectiveness in RPLS remains limited (41).

To the best of our knowledge, this is the first study aimed to systematically screen lipid metabolism related targets for RPLS. We conducted the profiles of somatic mutations and amplification in LMAGs, revealing potential targets that might be considered in RPLS. Considering the targets selected using the gene alteration profile might not be functionally significant, prognostic efficiency in OS and DFS and immune correlations were further evaluated to confirm the clinical relevance of the targets. ELOVL2 correlated with significantly prognosis and infiltration of APCs was identified. For instance, ELOVL2 was identified as an unique tissue-independent age-associated DNA methylation marker (42) and a specific superenhancer-associated gene implicated the LC-PUFA synthesis network as a critical metabolic dependency (43), and was associated with worsened patient survival in glioblastoma (44). In addition, ELOVL2 deficient indicated an increased infiltration of TH1/TH17 cells and a decrease of Treg (45). Thus, although basic and clinical investigation is further required, the potential of ELOVL2 to be successful targets was consolidated in RPLS.

Lipid metabolism and synthesis is based on the normal function of endoplasmic reticulum (ER). Previous results indicated that the accumulation of free fatty acids in the ER would eventually lead abnormal protein overloading and chronic ER stress (46). Meanwhile, the reduction of PUFA synthesis upon ELOVL2 decreasing can affect cellular metabolic homeostasis and mitochondrial energy metabolism (47). Unexpected, we found significantly activation of glucose metabolism pathway with ELOVL2 deficiency, such as gluconeogenesis, pentose phosphate pathway, pentose and glucuronate interconversions, fructose and mannose metabolism. This finds indicated that ELOVL2 deficiency may induce a switch in metabolism from the tri-carboxylic acid cycle to glycolysis, which eventually produces more reactive oxidative species (ROS) and causes oxidative stress. In addition, more mannose type O-glycan biosynthesis and lysine degradation was also found in the overexpression of ELOVL2. As the key regulator in PUFAs synthesis, more fatty acid elongation, but less arachidonic acid metabolism could be found in high group of ELOVL2, which was consistent with the recently published data (48). However, the relationship between abnormal expression phenotype of ELOVL2 and ER stress, mitochondrial dysfunction and cellular senescence still needs to be further studied.

Lipid metabolism reprogramming is a cretical marker of tumorigenesis and development. Previous study had indicated that a large number of lipid related genes underwent copy number amplification in the process of malignant development of RPLS (49). Therefore, it could be inferred that the amplification and high expression of lipid related gene-ELOVL2, was associated with malignant progression of RPLS with dismal prognosis. However, ELOVL2 displayed a heterogeneous role in the prognostic value in breast cancer (BC) (50) and glioma (44) (**Supplementary Figure 8**), respectively. In BC, ELOVL2 was hypermethylated and downregulated in the samples from tamoxifen resistance BC patients compared with those from tamoxifen-sensitive patients. Strikingly, in addition to having tumor suppressor activity, ELOVL2 was shown to recover tamoxifen sensitivity up to 70% in the MCF-7/tamoxifen resistance cells and in a xenograft mouse model (50). Furthermore, the depletion of ELOVL2 induced metastatic characteristics in BC cells via the SREBPs axis (51). In contrast, ELOVL2 depletion altered the phospholipid composition of the cell membrane by controlling fatty acid elongation, disrupting the structural characteristics of the cell membrane, and reducing EGFR signaling in glioblastoma cells (44). Taken together, we believed that abnormal lipid metabolism, including abnormal activation and inhibition, could both interfered with lipid metabolism reprogramming through different signal pathways in different types of cancers.

Since the tumor immune status is an another determinant of cancer associated efficacy, we further characterized the immune landscape in the different ELOVL2 subtypes. Interestingly, high ELOVL2 group is defined as immunological “cold” phenotype. In addition, the molecular signatures of this subtype is consistent with the immune status, indicating that patients with different immune signature may respond distinctly to immune therapy. To circumvent the poor immunogenicity of this subtype, targeted immune associated-biomarkers that reinvigorate the immune system by fascinating immune cell infiltrating may be a suitable option.

We demonstrated that RPLS patients could stratified into two LMSs with significant differences in molecular features and clinical prognosis. Patients with the LMS1 tumor had immune “hot” phenotype, whereas those with the LMS2 tumor had immune “cold” phenotype. Therefore, the lipid metabolism-associated molecular subtypes and risk model based on LMAGs were promising and complements the previous classification for RPLS.

With the prevalence of COVID-19 in the worldwide, the mRNA vaccine has highlighted its important strategic position, and greatly accelerating the development process of mRNA vaccine. Meanwhile, it also speeds up the research and development of mRNA cancer vaccine (52). The mRNA cancer vaccines represent promising novel method to treat malignancies with monotherapy or combination therapy (53). ELOVL2 is a potential target linking lipid metabolism to immune regulations for RPLS, specifically for patients with LMS2 tumors. However, given the function of ELOVL2 in lipid homeostasis and cellular homeostasis in normal cells, there is likely profound central tolerance against ELOVL2. On the contrast, ELOVL2 may be a target for small molecule inhibition for RPLS.

In summary, our study identified ELOVL2 as the potential effective target for RPLS, and patients with LMS2 tumor indicated an immune-excluded phenotype might benefit more from small molecule inhibition targerted ELOVL2.

## Data availability statement

The transcriptome data presented in the study are deposited in the Sequence Read Archive (SRA) repository, accession number PRJNA987378.

## Ethics statement

This study was reviewed and approved by the Institutional Review Board of Zhongshan Hospital (ID: B2022-586R), Shanghai, China. Informed consent was obtained from all study participants. All studies were performed in accordance with the Declaration of Helsinki.

## Author contributions

Conception and design: ZW, LM, YZ, HT. Development of methodology: ZW, PT, PF, YH. Acquisition of data (acquired and managed patients, provided facilities, etc.): ZW, JW, YHZ, WL, YZ. Analysis and interpretation of data (e.g., statistical analysis, biostatistics, computational analysis): ZW, PT, TR, LM. Writing, review, and/or revision of the manuscript: ZW, LM. Administrative, technical, or material support (i.e., reporting or organizing data, constructing databases): ZW, LM, HT. Study supervision: LH, LM, YZ, HT. All authors read and approved the final manuscript.

## Glossary

**Table:**

APC	Antigen presenting cells
AUC	Area under the curve
CAFs	Cancer associated fibroblasts
CNAs	Copy number alterations
COVID-19	*Corona virus disease 2019*
DCs	Dendritic cells
DDLPS	Dedifferentiated liposarcoma
DEG	Differential expression gene
DFS	Disease-free survival
ELOVL2	Elongation of very-long-chain fatty acids 2
ER	Endoplasmic reticulum
GEO	Gene expression omnibus
GO	Gene ontology
GSEA	Gene set enrichment analysis
GSE	GEO Series
GTEx	Genotype-tissue expression
ICD	Immunogenic cell death
IHC	Immunohistochemistry
KEGG	Kyoto encyclopedia of genes and genomes
LASSO	Least absolute shrinkage and selection operator
LC-PUFA	Long chain polyunsaturated fatty acids
LMAGs	Lipid metabolism-associated genes
LMS	Lipid metabolism subgroups
MCP-counter	Microenvironment cell populations-counter
MDSC	Myeloid-derived suppressor cells
mRNA	Messenger ribonucleic acid
MHC-II	Major histocompatibility complex-II
MSigDB	The molecular signatures database
M2-TAM	M2 T*umor-associated macrophage;*
NKT	Natural killer T cell
NR1H4	nuclear receptor subfamily 1 group H member 4
OS	Overall survival
PBMC	Peripheral blood mononuclear cell
PCA	Principal component analysis
PD-1	Programmed cell death protein 1
PD-L1	Programmed cell death protein ligand 1
PLCG1	Phospholipase C gamma 1
PPI	Protein-protein interaction networks
ROC	R*eceiver operating characteristic curve*
RCD	Regulated cell death
ROS	Reactive oxidative species
RPLS	Retroperitoneal liposarcoma
SARC	Sarcoma
sc-RNA-seq	single-cell RNA-sequencing
SD	Standard deviation
SPSS	*Statistical product and service solutions*
STRING	Search tool for the retrieval of interacting genes
TCGA	The cancer genome atlas
Tem	Effective memory T cell
TF	Transcription factor
Th1	*T helper cell 1*
Th17	*T helper cell 17*
TIME	Tumor immune microenvironment
TIMER	Tumor immune estimation resource
TIP	Tracking tumor immunophenotype
TLS	Tertitary lymphoid structures
TMB	Tumor mutation burden
TOM	Topological overlap matrix
TPM	Transcripts per kilobase of exon model per million mapped reads
Treg	*Regulatory T cells*
WDLPS	Well-differentiated liposarcomas
WGCNA	Weighted correlation network analysis.
