# Supplementary material for: Insight of a lipid metabolism prognostic model to identify immune landscape and potential target for retroperitoneal liposarcoma
Source: Front Immunol. 2023 Jul 6;14:1209396. doi: 10.3389/fimmu.2023.1209396 (PMC10359070; doi:10.3389/fimmu.2023.1209396)
Supplement: Supplementary file 1 [file DataSheet_1.docx]

**Supplementary Figures and Tables**

**
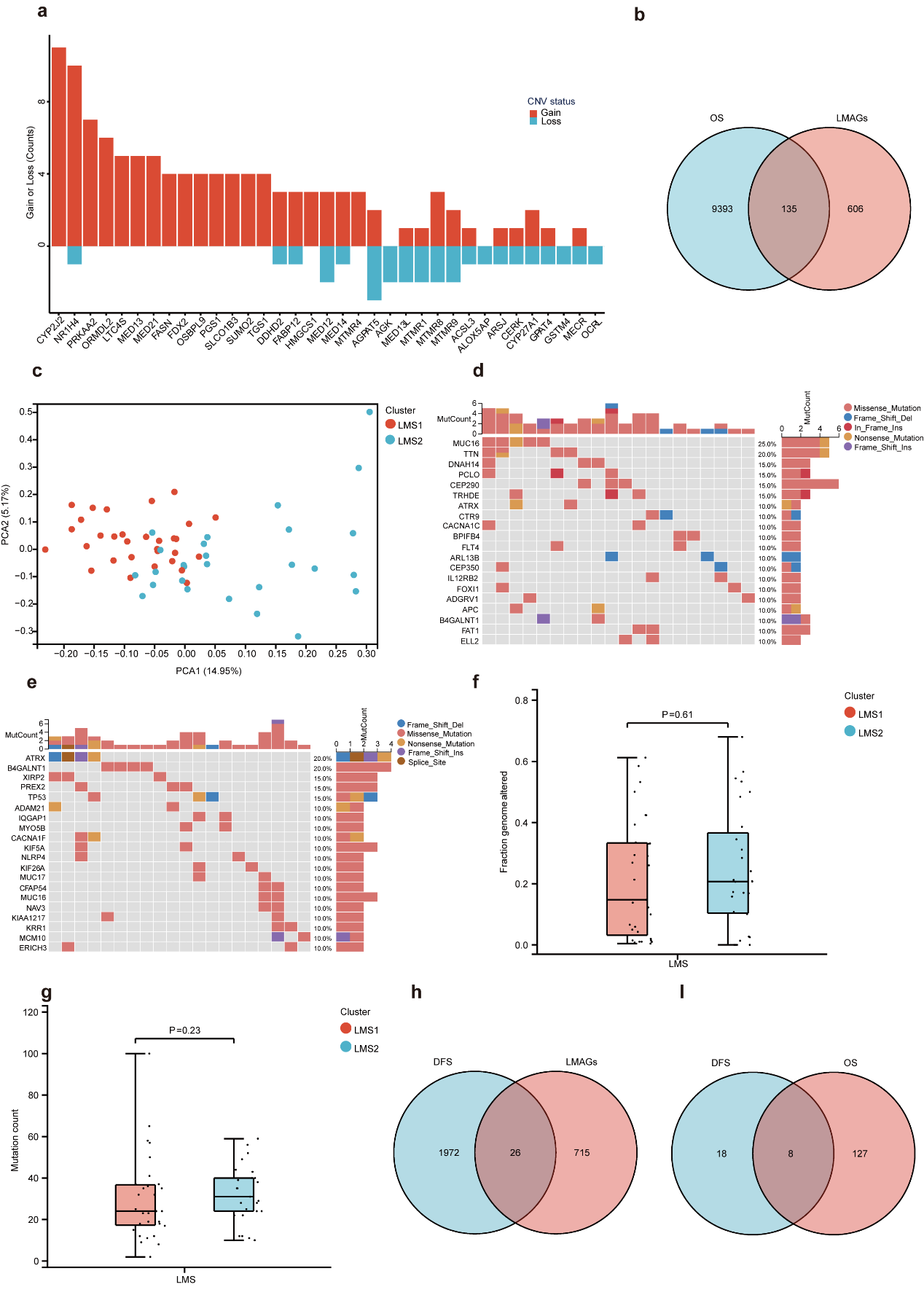
**

**Supplementary Figure 1. Identification of multi-omics landscape and prognostic LMAGs in RPLS.**

1. The CNV frequency of LMAGs in cohort-TCGA. (b) Venn diagram identifing 135 overlapping prognostic LMAGs. (c) Principal component analysis (PCA) distinguished two LMSs. (d-e) The landscape of the genomic alteration in the two LMSs. (f-g) The comparison of fraction genome altered and the number of mutation count evaluated in LMS1 and LMS2. (h-i) Venn diagram identifing 26 overlapped relapse LMAGs. Venn diagram identifing 8 overlapped OS-related and DFS-related LMAGs.


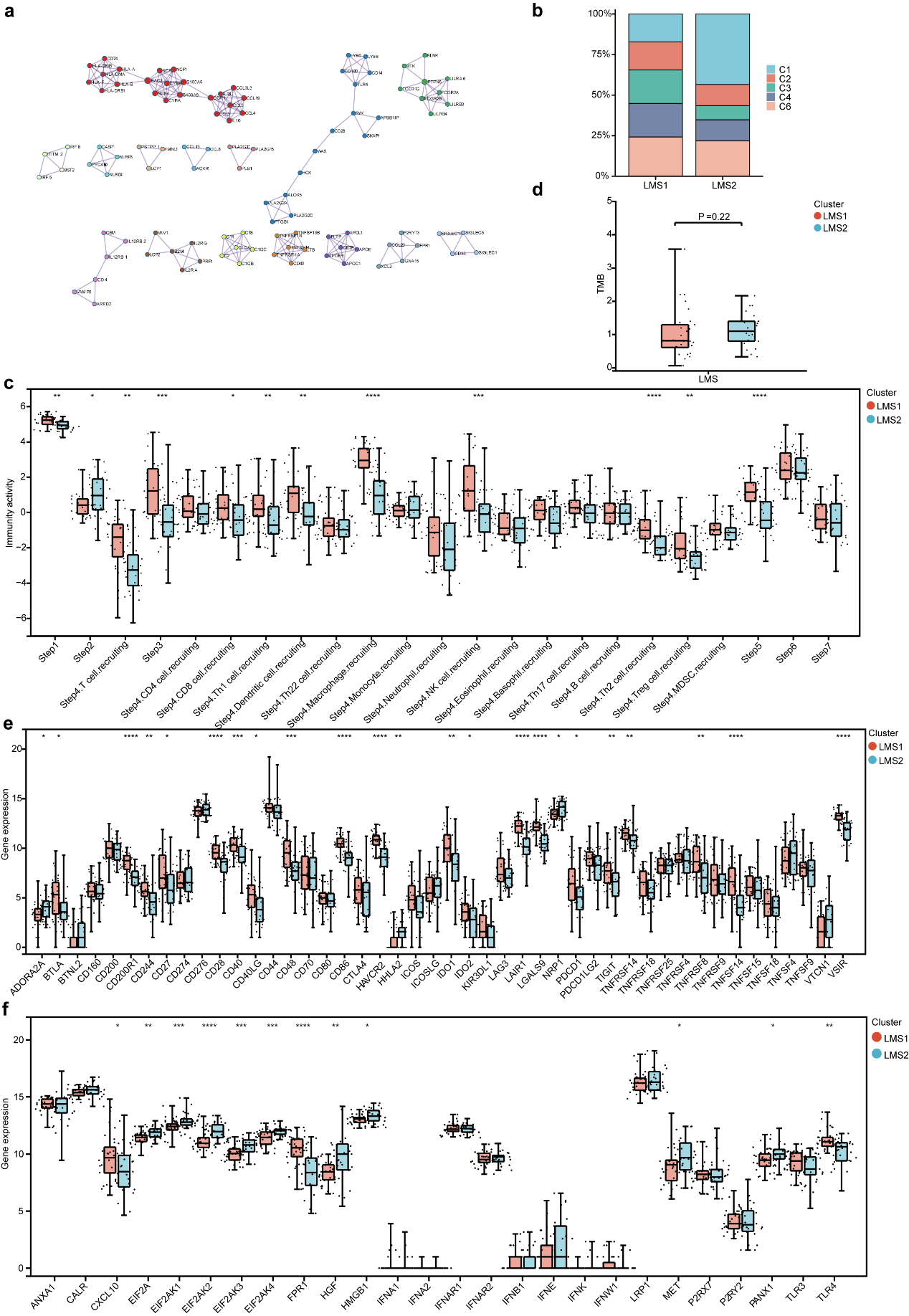


**Supplementary Figure 2. Heterogeneous functional enrichment and immune landscape in LMAGs subtypes.**

1. Sub models were identified by Matescape PPI analyses. (b) Distribution of individual immune categories between LMS1 and LMS2. (c) Distribution of immune activity scores between LMS1 and LMS2. (d) The comparison of Tumor mutational burden (TMB) between LMS1 and LMS2. (e) Difference in the expression of ICPs between LMS1 and LMS2. (f) The comparison of ICD modulators between LMS1 and LMS2. ns, not significant, * P < 0.05, ** P < 0.01, *** P < 0.001, and **** P < 0.0001.


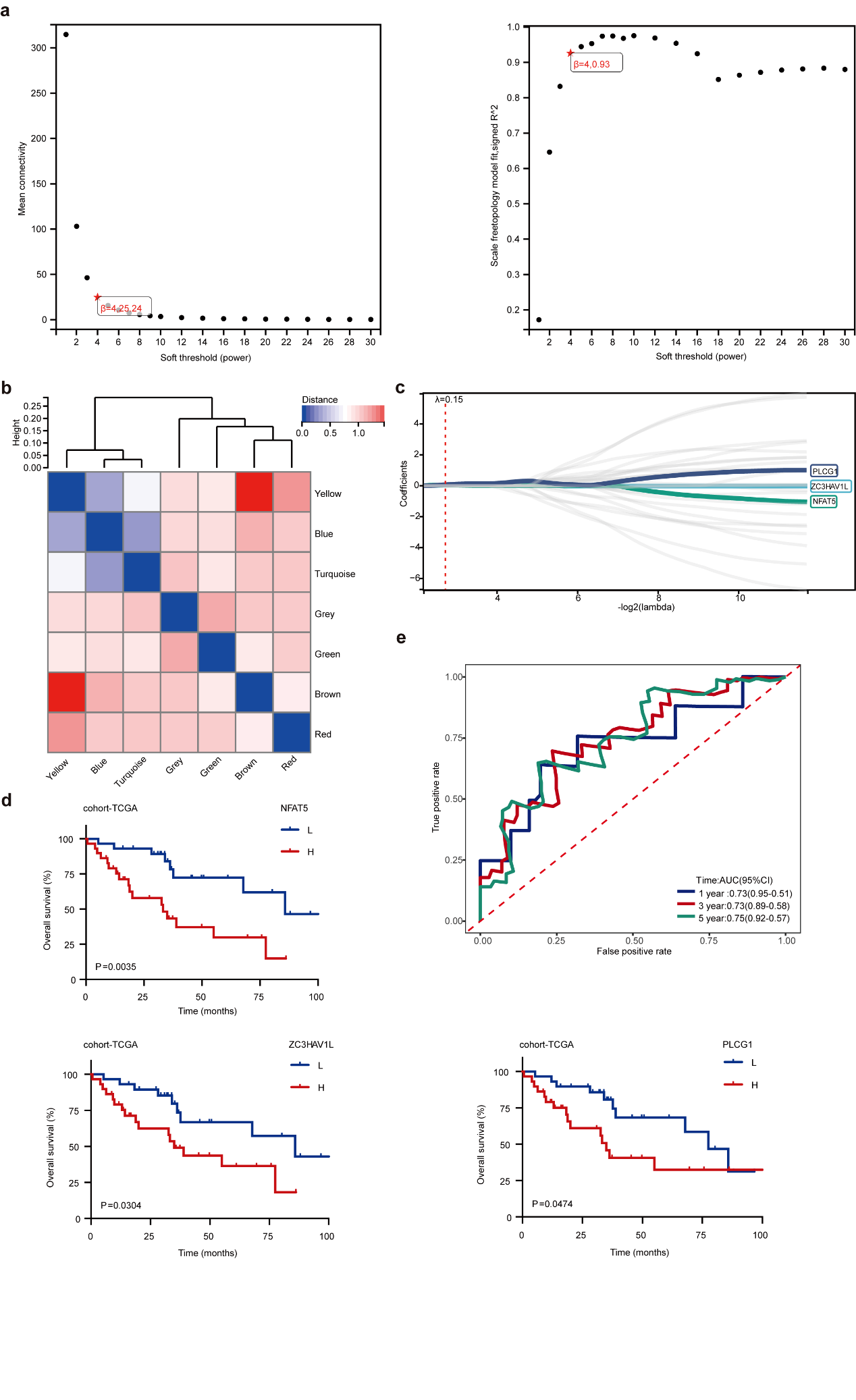


**Supplementary Figure 3.** **Identification of immune gene co-expression modules and immune hub genes of RPLS.**

(a-b) Gene co-expression network analysis based on the immune-related genes. (c) LASSO coefficient profiles of three selected immune hub genes in the 10-fold cross-validation. (d) Kaplan-Meier curves showing OS of RPLS stratified on the basis of ZC3HAV1L, NFAT5 and PLCG1 expression levels. (e) Time-dependent ROC curve of the risk model.

**
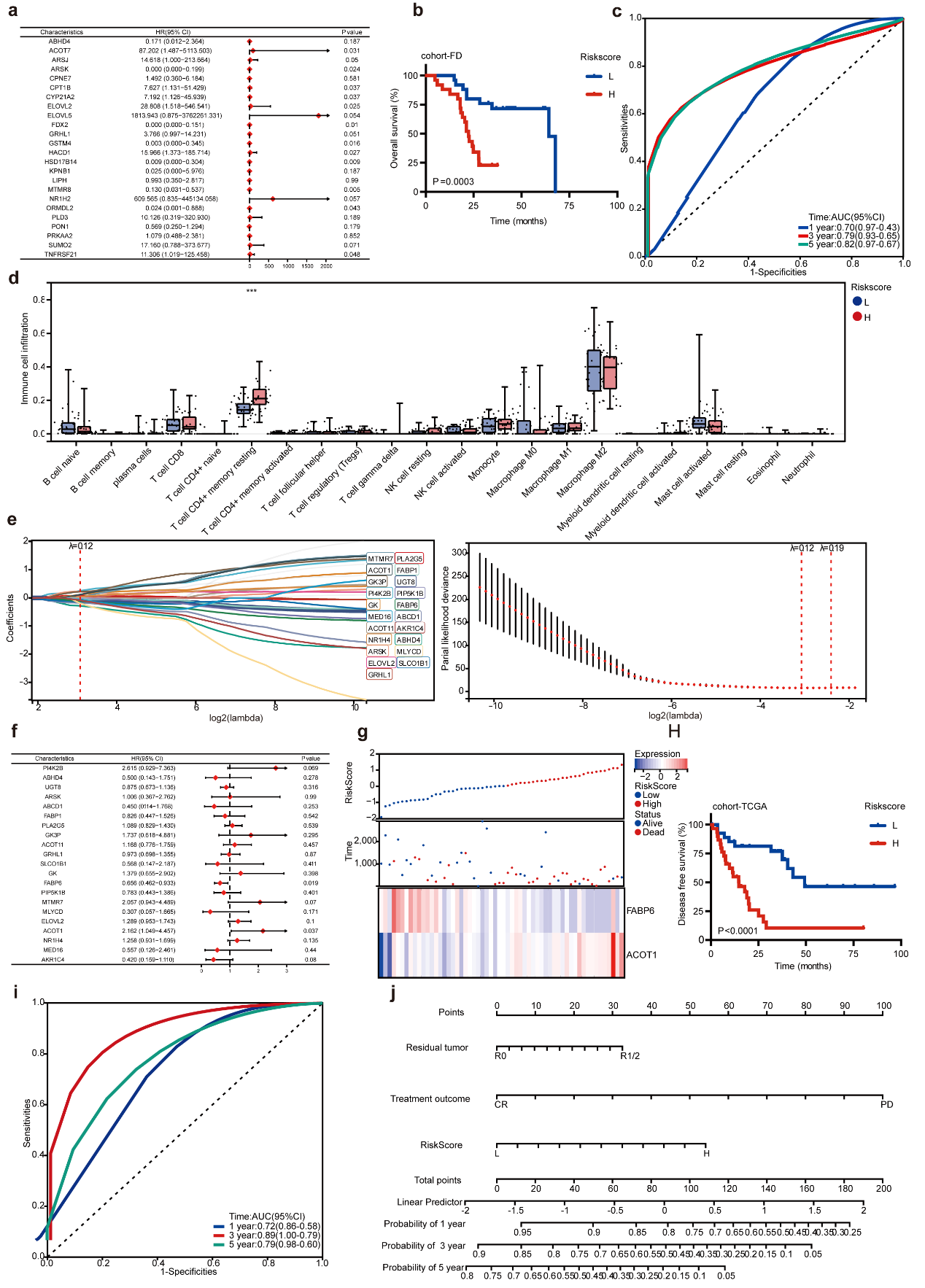
**

**Supplementary Figure 4. Development of survival and relapse risk models and nomograms based on LMAGs.**

1. Multivariate Cox analysis for further screening of 24 OS-related LAMGs. (b) Kaplan-Meier curve showing OS for two risk-score groups in cohort-FD. (c) Time-dependent ROC curve of the risk model in cohort-FD. (d) The comparison of 22 immune cells between LMS1 and LMS2. (e) LASSO analysis with minimal lambda. (f) Forest maps depicting prognosis prediction value of 21 DFS-related LAMGs. (g) Distribution of survival status and risk score of RPLS patients in the high and low risk groups, heatmap illustrating the expression of the two candidate genes in the two groups. (h) Kaplan-Meier survival curve of DFS for two risk-score groups in cohort-TCGA. (i) Time-dependent ROC curve of the risk model in cohort-TCGA. (J) Nomogram predicting DFS for RPLS. *** P < 0.001.


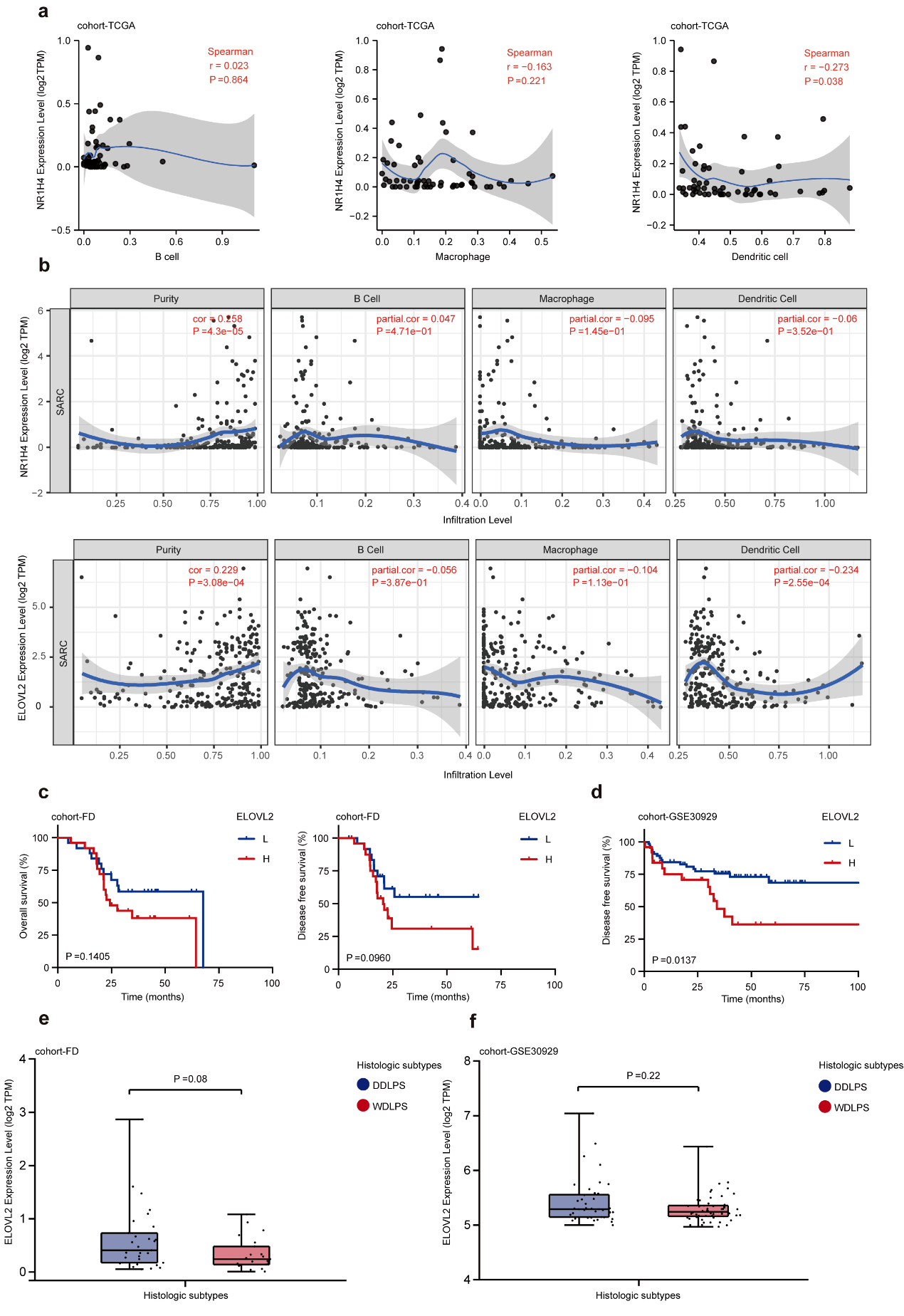


**Supplementary Figure 5.** **Identification of lipid metabolism-associated tumor antigens.**

Identification of tumor antigens associated with APCs. Correlation between NR1H4 expression and infiltration of macrophages, dendritic cells and B cells in cohort-TCGA. (b) Correlation between ELOVL2/NR1H4 expression and infiltration of macrophages, dendritic cells and B cells TCGA-SARC (n=264). (c) Kaplan-Meier survival curves of OS and DFS for ELOVL2 expression in cohort-FD. (d) Kaplan-Meier survival curves of DFS for ELOVL2 expression in cohort-GSE30929. (e) The comparison of ELOVL2 expression between DDLPS and WDLPS in cohort-FD and cohort-GSE30929.


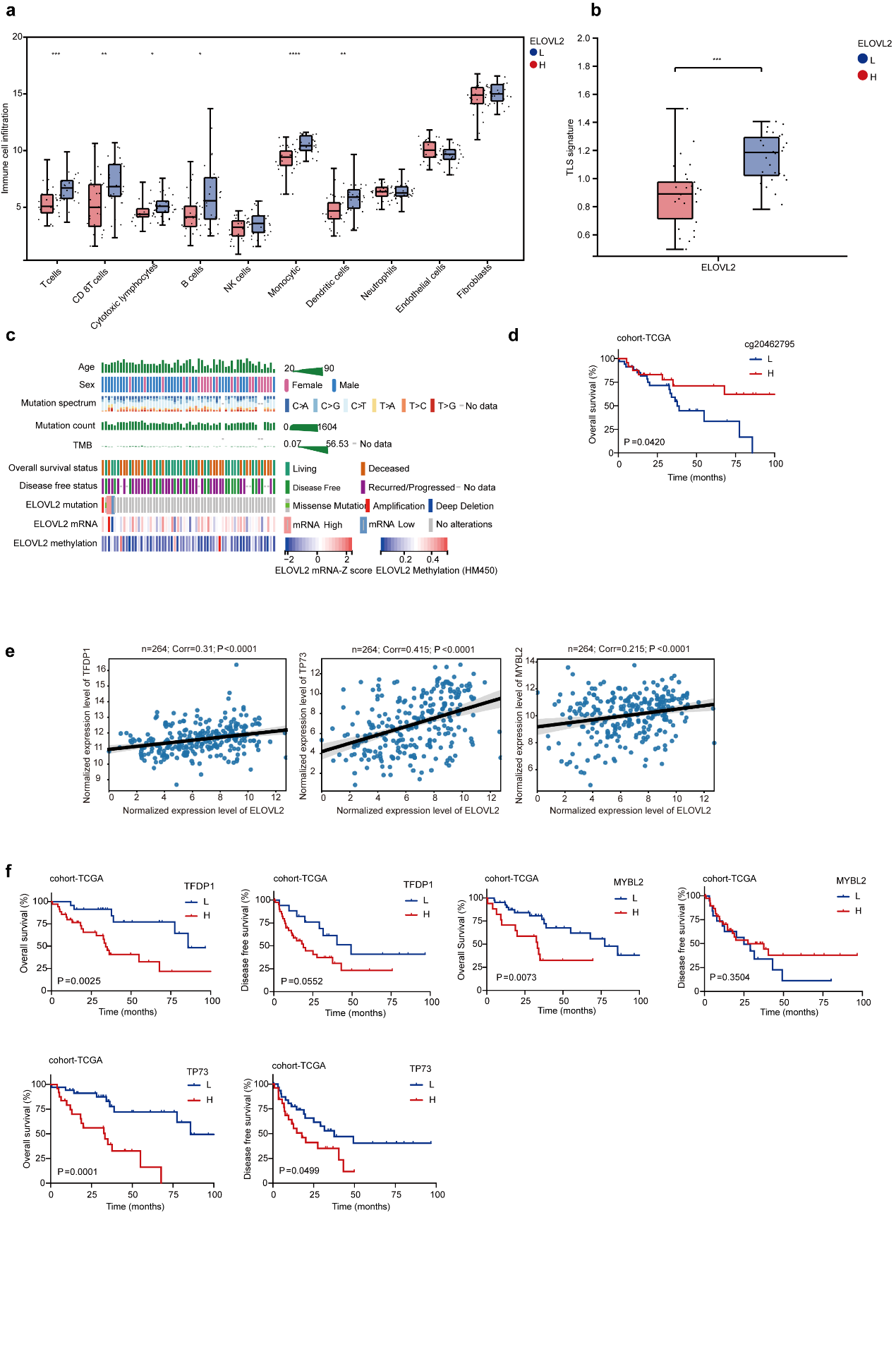
**Supplementary Figure 6. ELOVL2 dominated lipid metabolism reprogramming.**

1. The comparison of 10 types of immune cells between ELOVL2^high^ and ELOVL2^low^ groups. (b) The comparison of TLS signature according to ELOVL2 expression. (c) The genome and epigenome landscape of ELOVL2 in cohort-TCGA. (d) Kaplan-Meier survival curves of OS for cg20462795 in cohort-TCGA. (e) Correlation analyses between the expression levels of ELOVL2 and TFDP1,TP73, MYBL2 in TCGA-SARC (n=264). (f) Kaplan-Meier curves showing OS and DFS of TFDPI, MYBL2 and TP73 in cohort-TCGA. ns, not significant, * P < 0.05, ** P < 0.01, *** P < 0.001, and **** P < 0.0001.

**
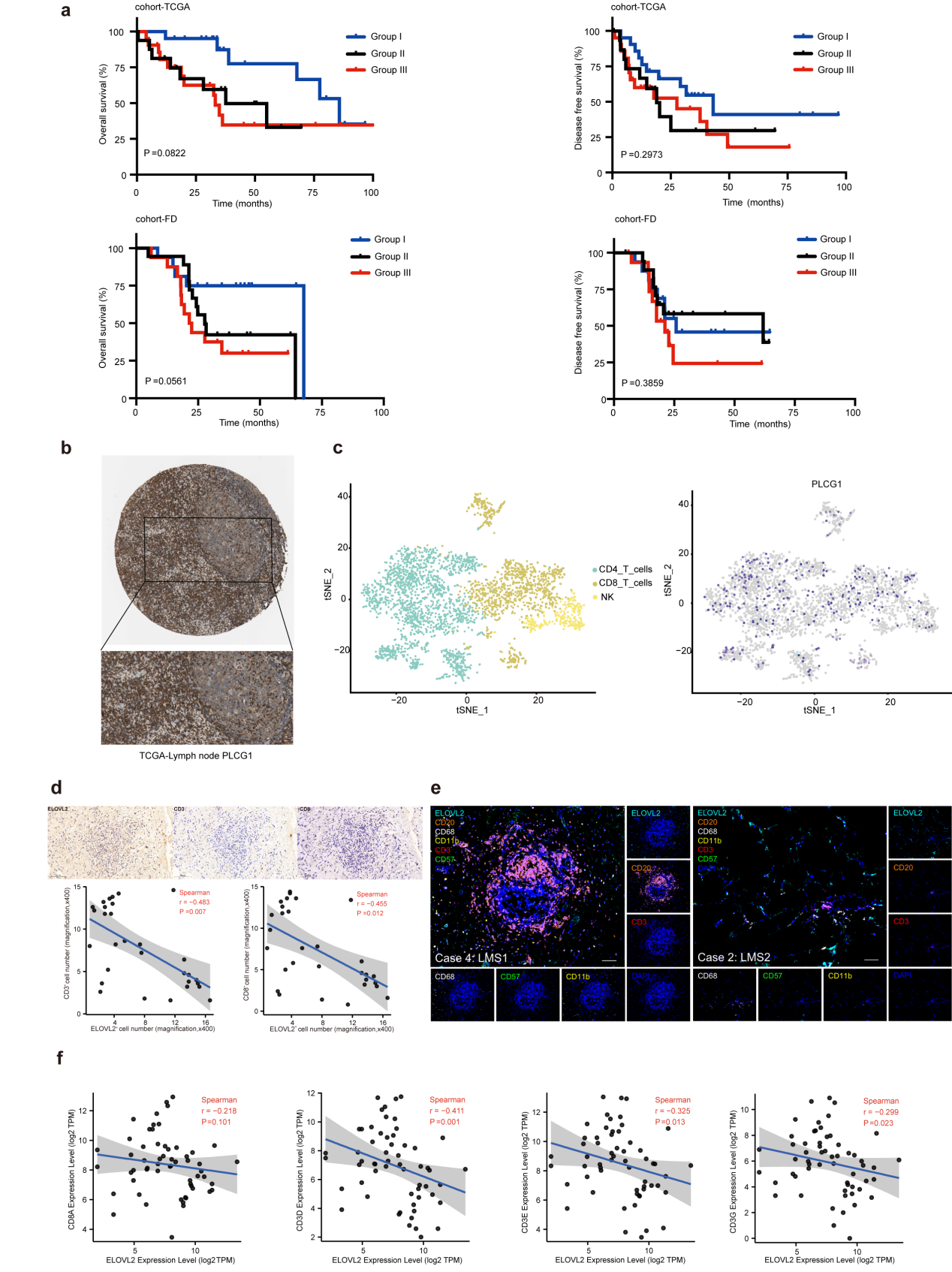
**

**Supplementary Figure 7. ELOVL2 dominated lipid metabolism reprogramming and executive TIME affect prognosis in RPLS.**

(a) Kaplan-Meier survival curves of OS and DFS for ELOVL2 and PLCG1 expression in cohort-TCGA and cohort-FD. (b) Representative IHC images showing the staining for PLCG1 in TCGA-lymph node (Scale bar: 50 μm). (c) t-SNE plot showing of the overview of PLCG1 in T cells. (d) Representative IHC images showing the staining for ELOVL2, CD3 and CD8 in RPLS. (e) Representative mIHC images show the positivity of CD3, CD8, CD20, CD11b, CD68, and ELOVL2 in case 4 and case 2. Scale bar, 100 um. (f) Correlation analyses between the transcriptomic expression of ELOVL2 and CD8A, CD3D, CD3E and CD3G in cohort-TCGA.

**
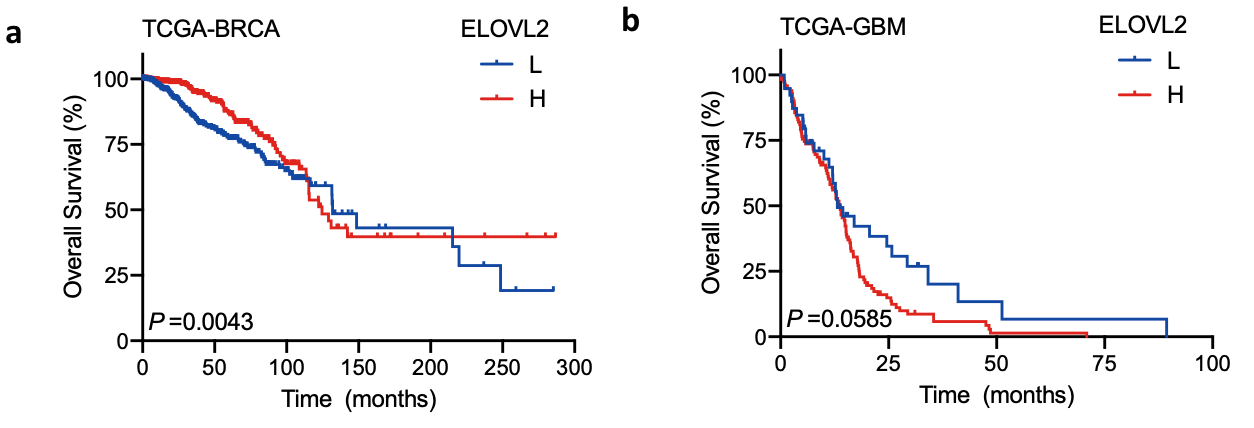
**

**Supplementary Figure 8. ELOVL2 displayed a heterogeneous role in the prognostic value in breast cancer (BC) and glioma (GBM).**

(a) Kaplan-Meier survival curves of OS for ELOVL2 mRNA expression in TCGA-BC. (b) Kaplan-Meier survival curves of OS for ELOVL2 mRNA expression in TCGA-GBM.

**Supplementary Tables**

**Supplementary Table 1.** **Clinical characteristics of liposarcome patients in cohort-FD (n=50) and cohort-TCGA (n=58).**

|  | cohort-FD | | cohort-TCGA | |  |
| --- | --- | --- | --- | --- | --- |
| Characteristics | n=50 | % of total | n=58 | % of total | P |
| Gender |  |  |  |  | 0.9580 |
| Male | 33 | 66% | 38 | 65.5 |  |
| Female | 17 | 34% | 20 | 34.5 |  |
| Age |  |  |  |  | 0.0151 |
| <60 | 28 | 56% | 19 | 32.8 |  |
| ≥60 | 22 | 44% | 39 | 67.2 |  |
| Histologic subtypes |  |  |  |  | <0.0001 |
| WDLPS | 21 | 42% | 0 | 0 |  |
| DDLPS | 29 | 58% | 58 | 100 |  |
| Tumor size |  |  |  |  | 0.8358 |
| <20 | 32 | 64% | 36 | 62.1 |  |
| ≥20 | 18 | 36% | 22 | 37.9 |  |
| Tumor number |  |  |  |  | 0.5532 |
| Single | 34 | 68% | 35 | 60.3 |  |
| Multiple | 16 | 32% | 21 | 36.2 |  |
| Complete resection |  |  |  |  | 0.0253 |
| Yes | 34 | 68% | 26 | 44.8 |  |
| No | 16 | 32% | 30 | 51.7 |  |
| FNCLCC |  |  |  |  | NA |
| 1 | 14 | 28% | NA | NA |  |
| 2 | 23 | 46% | NA | NA |  |
| 3 | 13 | 26% | NA | NA |  |
| Recurrence |  |  |  |  | 0.7416 |
| Yes | 26 | 52% | 32 | 55.2 |  |
| No | 24 | 48% | 26 | 44.8 |  |

**Supplementary Table 2.** **Clinical characteristics of liposarcome patients in cohort-SC-RNA seq(n=4) .**

|  | **cohort-SC-RNA seq** | |
| --- | --- | --- |
| Characteristics | n=4 | % of total |
| Gender |  |  |
| Male | 1 | 25% |
| Female | 3 | 75% |
| Age |  |  |
| <60 | 3 | 75% |
| ≥60 | 1 | 25% |
| Histologic subtypes |  |  |
| WDLPS | 0 | 0% |
| DDLPS | 4 | 100% |
| Tumor size |  |  |
| <20 | 1 | 25% |
| ≥20 | 3 | 75% |
| Tumor number |  |  |
| Single | 2 | 50% |
| Multiple | 2 | 50% |
| Complete resection |  |  |
| Yes | 4 | 100% |
| No | 0 | 0% |
| FNCLCC |  |  |
| 1 | 0 |  |
| 2 | 3 | 75% |
| 3 | 1 | 25% |
| LMS |  |  |
| LMS1 | 2 | 50% |
| LMS2 | 2 | 50% |

**Supplementary Table 3. Anti-human Antibodies Used in Multiplexed immunohistochemistry.**

| **Ant­ibody** | **Catalogue or clone** | **Company** |
| --- | --- | --- |
| CD3 | ab16669, SP7 | Abcam |
| CD20 | ab9475, L26 | eBioscience |
| CD68 | ab955, KP1 | Abcam |
| CD57 | ab233872, NK-1 | Abcam |
| CD11b | Ab133357, EPR1344 | Abcam |
| ELOVL2 | 20308-1-AP | Proteintech |

**Supplementary Table 4.** **The workflow chart systematically evaluated and depicted the whole study process.**

**Transcriptome analysis**

**scRNA-Seq**

**analysis**

**DNA methylation**

**Transcriptome analysis**

**135 prognosis-associated**

**LMAGs (n=741)**

**AMP genes & Mutated genes &**

**OS gene & DFS genes & LMAGs**

**Consensus**

**clustering**

**LASSO regression and multivariate Cox regression**

**Survival**

**analysis**

**Genome**

**analysis**

**Immune**

**status**

**DEGs**

**Estimated**

**GO**

**13 LMAGs signature**

**2 LMAGs signature**

**Construction of nomogram**

**APCs correlations**

**Antigen identification**

***ELOVL2***

**IHC**

**cohort-GSE30929 and cohort-FD**

**26 relapse-associated**

**LMAGs (n=741)**

**LASSO regression and multivariate Cox regression**

**2 candidate genes:**

***ELOVL2* and *NR1H4***

**TLS**

**WGCNA**

**ICP**

**ICD**

**MCP**

**Cibersort**

**TIP**

**KEGG**

**PPI**

**CNV analysis**

**Mutation analysis**

**Risk score constructed**

**Survival analysis**

**Correlation of LMAGs**

**risk and TIME**

**Construction of nomogram**

**Risk score constructed**

**Survival analysis**

**cohort-TCGA**

**(n=58)**
